# Selection of Early Life Codons by Ultraviolet Light

**DOI:** 10.1021/acscentsci.4c01623

**Published:** 2025-01-08

**Authors:** Corinna L. Kufner, Stefan Krebs, Marlis Fischaleck, Julia Philippou-Massier, Helmut Blum, Dominik B. Bucher, Dieter Braun, Wolfgang Zinth, Christof B. Mast

**Affiliations:** † Harvard-Smithsonian Center for Astrophysics, Department of Astronomy, Harvard University, 60 Garden Street, Cambridge, Massachusetts 02138, United States; ‡ Laboratory for Functional Genome Analysis, Gene Center, Ludwig-Maximilians-University Munich, Feodor-Lynen-Straße 25, 81377 Munich, Germany; § Department of Chemistry, Technical University of Munich, Lichtenbergstr. 4, 85748 Garching, Germany; ∥ Systems Biophysics, Ludwig-Maximilians-University Munich, Amalienstr. 54, 80799 Munich, Germany; ⊥ Biomolecular Optics and Center for Integrated Protein Science, Ludwig-Maximilians-University Munich, Öttingenstrasse 67, 80538 Munich, Germany

## Abstract

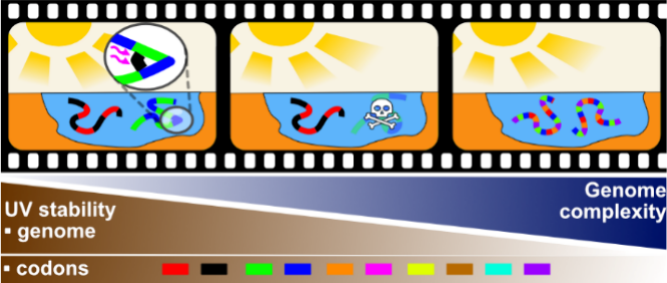

How life developed in its earliest stages is a central but notoriously difficult question in science. The earliest lifeforms likely used a reduced set of codon sequences that were progressively completed over time, driven by chemical, physical, and combinatorial constraints. However, despite its importance for prebiotic chemistry, UV radiation has not been considered a selection pressure for the evolution of early codon sequences. In this proof-of-principle study, we quantified the UV susceptibility of large pools of DNA protogenomes and tested the timing of evolutionary incorporation of codon sequences using a Monte Carlo method utilizing sequence-context-dependent damage rates previously determined by high throughput sequencing experiments. We traced the UV-radiation selection pressure on early protogenomes comprising a limited number of codon sequences to late protogenomes with access to all codons. The modeling showed that in just minutes under early sunlight, the choice of the first codons determined whether most of the protogenomes remained intact or became damaged entirely. The results correlated with earlier chemical models of the evolution of the genetic code. Our results show how UV could have played a crucial role in the evolution of the early genetic code for a DNA-based genome and provide the concept for future RNA-based studies.

## Abstract 2

Assessing the UV susceptibility of early DNA protogenomes by their use of codon sequences correlates with established amino acid chronologies, supporting the UV compatibility of early life.

## Introduction

Solving the question of the origins and development of early life on Earth requires multifaceted approaches. While bottom-up models such as the RNA world hypothesis investigate the onset of minimal life forms built around RNA as a universal information storage and functional enzyme,^1^ top-down models through phylogenetic trees approach the last universal common ancestors (LUCA) from the opposite direction. In such extrapolations of LUCA, the central dogma of molecular biology and its genetic code must already have been implemented,^2^ creating a gap between top-down and bottom-up approaches that must explain how this proto-life started to use the interplay of DNA, RNA^3−5^ and proteins through the genetic code under the harsh conditions of the early Earth.^6−8^

Under this premise, it was argued that the genetic code likely did not address all possible codon sequences from the start. Instead, it included them step by step along a specific chronology guided by combinatorial arguments or chemical and physical selection pressures on the early Earth.^9−11^ However, the impact of ultraviolet (UV) radiation on protogenomes or the order of appearance of utilized codon sequences has not been studied to our knowledge despite its significant role in prebiotic chemistry.

In promising surficial scenarios for emerging life, such as shallow lakes,^8^ UV irradiation likely acted as a significant selection pressure. It has been discussed as an essential source of energy to drive prebiotic chemical reactions that generate the building blocks of life, such as nucleic acids, peptides, and lipids,^6,12−17^ as well as a selector for prebiotic organics, including the canonical nucleobases^18^ or RNA.^19−22^

**Figure 1:**
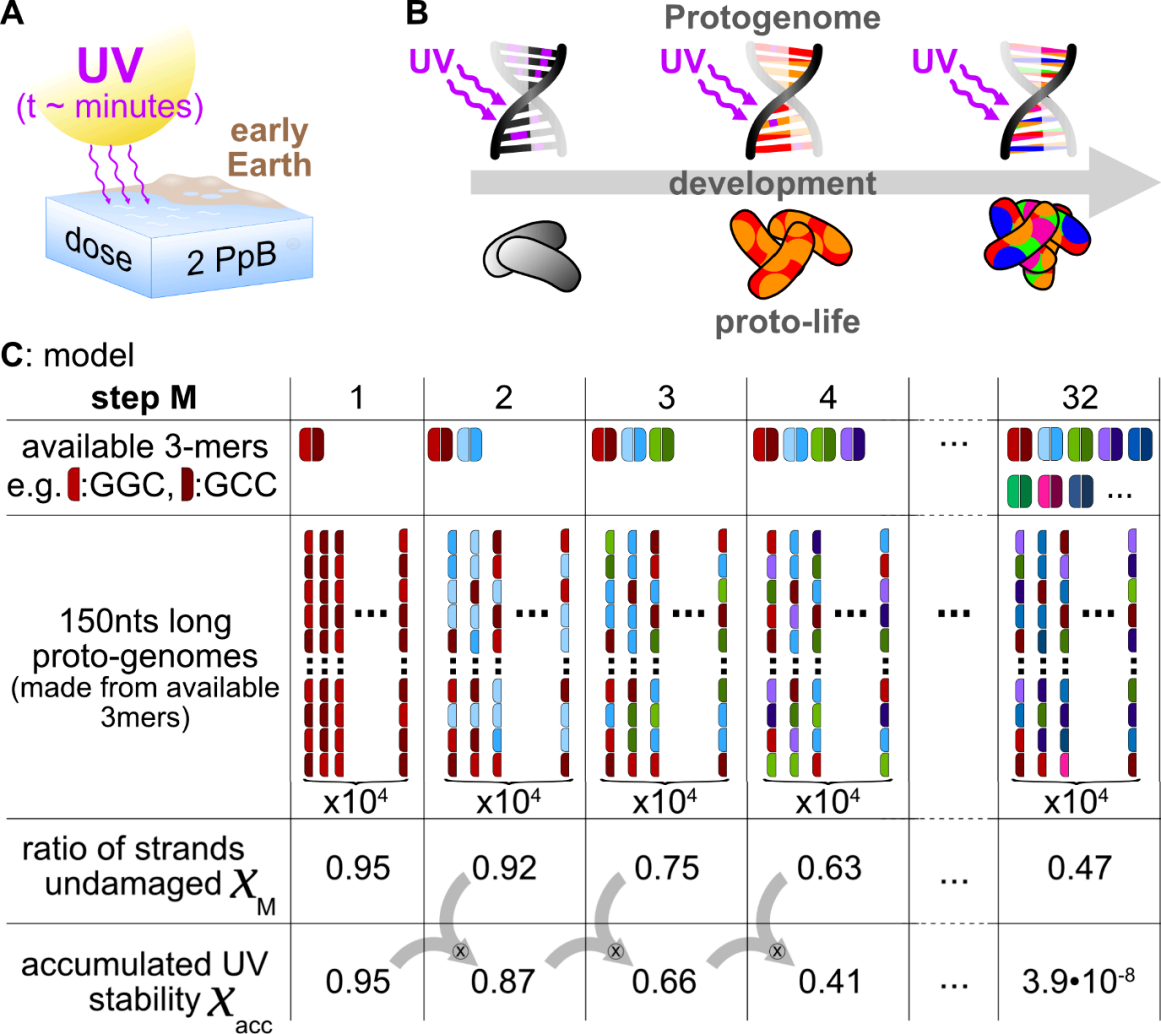
Surface scenario for UV-based selection of the first life forms. (A) The early Earth was exposed to intense UV radiation due to the lack of an ozone layer. However, at the surface of the earth irradiation is so strong, that each base absorbs two UV photons within less than 4 min, posing severe selection pressure for early genomes. The irradiation dose is stated in PpB, i.e., the photons per base. (B) The first life was likely simpler and had access to fewer amino acids than today. This restricted protogenome apparently did not use all codon sequences, leading to reduced sequence patterns. Over time, life produced more complex proteins with all proteogenic amino acids, utilizing all available codon sequences. (C) Model for the UV-influence on codon evolution of a hypothetical DNA protogenome (150-mer): In simple proto-lifeforms, only a few amino acids and a small part of the genetic code were used early on (step number M is small). Only proto-genomes using UV-stable codons would survive here. Throughout its development, newly added codons at higher step numbers M change the UV stability of the protogenome until all 32 codon pairs (64 triplets) are used in the genome. The sequence of added codons is termed codon chronology. The product  of all ratios of undamaged strands gives a metric for the UV stability of a specific codon chronology, determining the accumulated UV Stability χ_*acc*_.

At the same time, the substantial influx of UV photons below 300 nm due to the missing ozone layer posed a significant threat to polynucleic acids at the surface, even after short exposures (see SI Appendix, section 1 Figure 1A). The resulting photolesions such as cyclobutane-pyrimidine-dimers or 6–4 photoproducts^23,24^ were shown to impact the duplex formation of polynucleic acids^25^ and strand stability.^18,26,27^ Without access to modern repair enzymes, these effects would likely have hindered the copying of protogenomes. Early life would, therefore, have benefited from the provision of UV-stable protogenome sequences through a suitable choice of the first codon sets (Figure 1B).

This work quantifies the UV susceptibility of protogenome pools based on the comprising codon sequences used by early life. Starting from the simplest protogenomes containing only a few different codon sequences, we focus on how UV stability changes when additional codon sequences are involved and compare the resulting codon chronologies with established models of genetic code evolution.

Since little evidence is available from this early period in the development of life, this proof-of-concept study aims to show how a minimum set of presumptions and experimentally obtained data on UV susceptibility can be used to draw conclusions about the development of the genetic code. By selecting suitable codons, early life could therefore have ensured that protogenomes were as UV-stable as possible. The hypothesis that early genomes initially use only a few codon sequences and thus do not cover the entire sequence space, also called sequence bias, is supported by the sequence dependence of early polymerization^28^ and replication schemes,^29−31^ yielding highly biased sequence pools.

Our model uses a Monte Carlo approach fed by experimentally determined sequence-context-dependent trimer damage rates that predict the UV lesion formation for large pools of 150 nucleotide-long DNA protogenomes under a fixed irradiation dose. Even for this short genome length and a moderate dose of two absorbed 266 nm photons per base, likely to be reached after only a few minutes on early Earth, we find a strong influence of UV irradiation. Depending on the sequence patterns that constitute the protogenome, situations occur where only a small part or almost all strands of the protogenome pool show UV damage.

Our choice of single-stranded DNA (ssDNA) as a protogenome model results from the fact that the complete sequence dependence of UV damage is only known for ssDNA.^32^ However, our results provide a basis for future analog studies for dsDNA or RNA, as ssDNA shows similar photophysical modifications, such as CPD damages.^33^ In addition, UV radiation would massively affect surface-near early life as soon as it uses DNA as a chemically stable genetic memory, as it accumulates significant UV damage over its long lifetime.

Given these constraints, our proto-genome model allows us to study the development of protogenomes under UV light as selection pressure and rank codon chronology models mentioned above^9−11^ concerning their compatibility with UV radiation. A high UV susceptibility of the protogenomes resulting from these chronologies would suggest that UV radiation may not have played a major role in the selection of the code. To the contrary, our results suggest an important role of UV light in the selection process of early life on Earth and pave the way for future RNA-based studies.

## Results

### Protogenome Model

We define the protogenome as the genetic repository of an early pre-LUCA life form with a specific sequence bias and assume that this sequence bias changes throughout the life form’s development. Due to the limited knowledge about the specifics of such a protogenome and its development, we approach this problem with a general model that allows us to obtain quantitative information about the UV stability of the protogenomes and their proposed developmental pathways (Figure 1C).

In our model, we do not consider the protogenome as a specific set of single sequences that are coupled to certain enzyme functions but as a large ensemble of 10^4^ strands with sequences randomly built from a common set of codon trimers. Without loss of generality, we fixed the length of the protogenome strands to 150 nucleotides, which is longer than the size of the genes encoding the most primitive proteins^34^ but still short enough to cover a statistically relevant number of sequences (see Figure S1).

As this is a proof-of-concept study, we focus on ssDNA as genetic material, since only for ssDNA the sequence dependence of UV lesion formation up to the second nearest neighbors and its effect on readout processes has recently been investigated.^32^

Irrespective of this choice of genetic storage, such early protogenomes would likely have comprised significant sequence biases resulting from minimal replication networks^30,31^ and the limited availability of amino acids and their linked codon sequences.^9−11^ We mimic this protogenome sequence bias and its development in 32 distinct steps *M* = 1,..,32 by starting in the first step from a maximally restricted pool of 150-mers, which are randomly concatenated from one codon-sequence (trimer) and its reverse complement (Figure 1C, *M* = 1, red blocks). Codon sequences and their reverse complements are assumed to co-occur, as they are inherently linked by templated replication.

Over time, the protogenome will include another trimer sequence and its reverse complement in a second step (Figure 1C, *M* = 2, blue blocks). This addition of codon sequence pairs is repeated until the complete sequence space is accessible through all possible 3^4^ = 64 trimers, equivalent to 32 trimer-pairs in the last developmental step (Figure 1C, *M* = 32, all colors). Each developmental step is characterized solely by the sequence patterns occurring in the protogenome and contains no further assumptions, such as the time scale on which these changes occur or the specifics of the replication process, thus remaining as generally valid as possible.

For each development step *M*, we calculate the formation of UV lesions on 10^4^ protogenome strands that are randomly built from the step-specific set of trimers. We implement the in-silico irradiation of the 10^4^ protogenomes using a Monte Carlo-type simulation as described below, using experimentally found trimer-damage rates (also see SIAppendix section 2). Thus, the fraction χ_*M*_ of intact protogenomes without any photodamage is obtained, which is a direct measure of the stability against UV irradiation at a given developmental step *M*. In this context, proto-life with a UV-sensitive protogenome (χ → 0) can be assumed to be more at risk of extinction than those with stable protogenomes (χ → 1), as UV damage is likely to lead to mutations or complete failure of replication processes.

In the example shown in Figure 1C, we obtain a ratio of undamaged strands of χ_*M*=1_ = 0.95 in the first step for 150-mers composed only of the highly UV-stable sequences GGC and GCC (red blocks). The ratio decreases with increasing diversity of possible sequence patterns (blocks of other colors) as base pairs that can form more photolesions are introduced. The order in which new trimer pairs are added is called the chronology (C). The total number of possible chronologies is too large for living systems to explore completely (ca. 32! ∼ 10^35^), as in each step, the newly added trimer pair can be selected among all pairs not yet used in previous steps. Therefore, the chronology implemented was either randomly chosen or predetermined by chemical or combinatorial boundary conditions, which, however, have so far been investigated without including UV radiation.^9−11^ Here, we close this gap, showing below how UV radiation affects codon development and present a chronology based purely on this selection pressure.

To compare the impact of UV irradiation on different codon chronologies C, we introduce its accumulated UV stability, χ_*acc*,*N*=32_(*C*)=∏_*M* = 1_^*N* = 32^χ_*M*_, which is formed from the product of the fractions of undamaged strands χ_*M*_ of each development step *M* in the chronology (Figure 1C, last row). The accumulated UV stability is a relative measure for realizing a specific chronology up to a step *N*. Here, the multiplication causes single steps with low UV stability to significantly impact the accumulated UV stability. Thus, a highly UV-susceptible protogenomes with each of its 10^4^ strands strongly damaged at some step *M*^′^ (χ_*M*^′^_ → 0) would lead to a vanishing accumulated UV stability of the whole protogenome chronology, rendering the survival of the associated life form unlikely. To obtain the respective fractions of undamaged strands χ_*M*_ through a Monte Carlo approach, the sequence-context-dependent damage rates for UV lesion formation must first be determined from experimental data, as described in the following.

### Context-Dependent Rates for Lesion Formation

**Figure 2:**
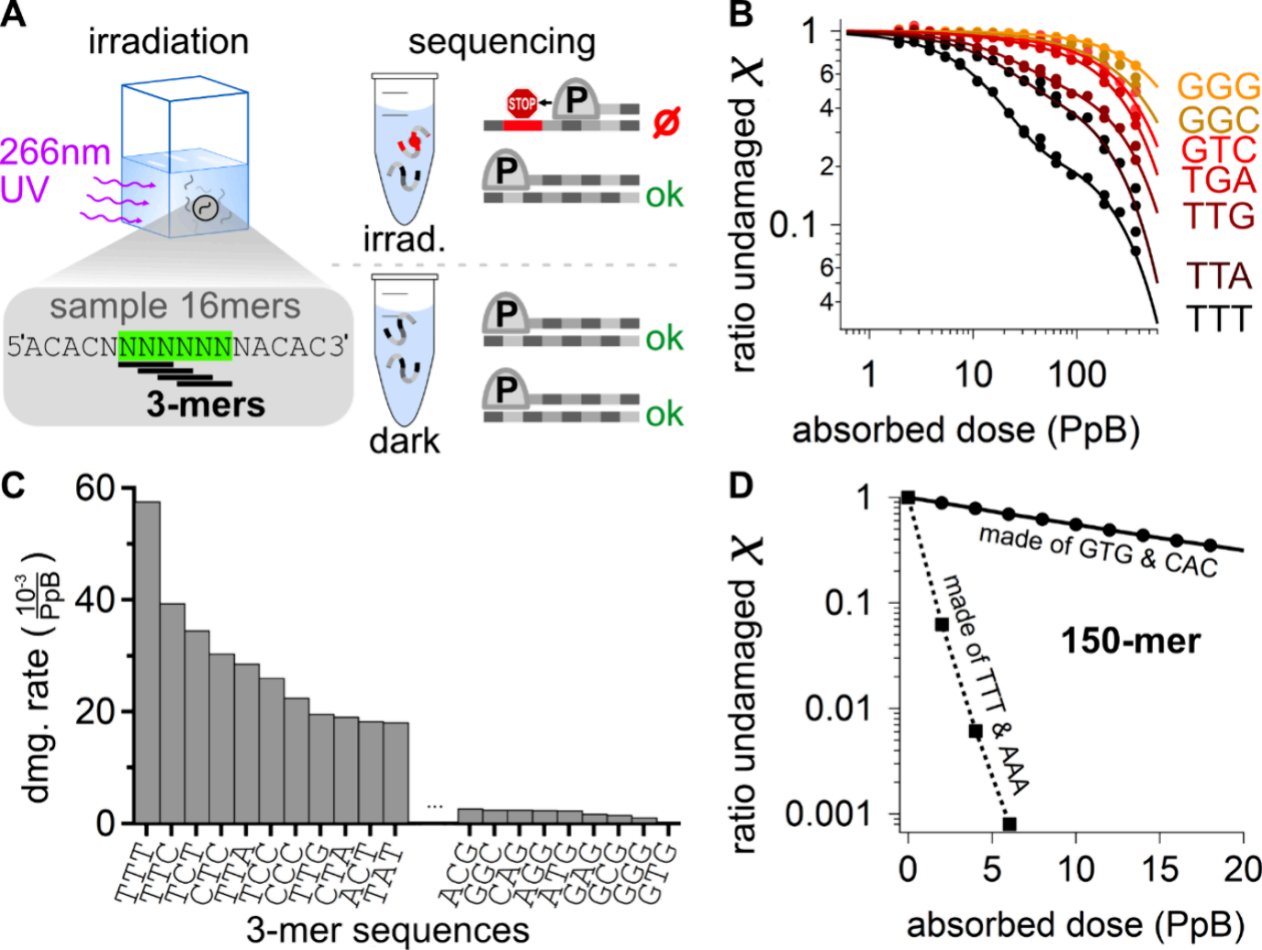
Measured UV susceptibility of DNA triplets and its impact on longer DNA strands. (A) A solution of d(ACAC-NNNNNNNN-ACAC) sequences with eight random canonical bases in the center (random 8mers) was irradiated at 266 nm. During irradiation, the solution is probed at 16 regular intervals and analyzed by next-generation sequencing. Damaged strands are not detected in sequencing due to efficient polymerase stalling.^32^ (B) Undamaged ratio χ of surviving 3mers within the random 8mers as a function of the absorbed dose in units of photons per base (PpB) with corresponding fit curves (lines). (C) Damage rates of selected 3mers, shown in (A), e.g., GGG, GGC, GTC. (D) Ratio of undamaged 150mer DNA strands as a function of the absorbed dose for two averaged exemplary proto-genomes (150mers) made from different codon pairs (symbols: Monte Carlo simulation, lines: exponential fit).

The trimer damage rates and sequence-context-dependent lesion formation were quantified by analyzing a previously measured data set from high throughput sequencing (HTS) of irradiated ssDNA samples.^32^ The experimental approach is shown in Figure 2A: A sample consisting of single-stranded DNA oligomers with a length of 16 bases and the sequence ACACNNNNNNNNACAC was irradiated at 266 nm in aqueous solution at neutral pH for increasing time intervals. The central eight bases of the oligomers consisted of randomized sequences, where N represents one of the four canonical nucleobases, dA, dT, dC, dG, and tag sequences consisting of the sequence d(ACAC) on both the 5′ and 3′ ends. The sample covered the 4^8^ (= 65536) possible octamer sequences of the central randomer part. The chosen wavelength was present in the UV irradiation on early Earth at significant intensities and excited the nucleobases near their absorption maximum to produce the most common types of UV lesions.^24,35^

After each irradiation interval, a small part of the sample was removed and analyzed by HTS. The sequencing library preparation used high-fidelity polymerases that stall efficiently upon reaching dimeric photolesions^36^ so that damaged strands are not amplified (Figure 2A, right). As a result, the number of reads of highly UV-susceptible sequences decreases with the irradiation dose. It allowed us to determine the ratio of undamaged 16-mer strands of each sequence by normalization against a nonexposed control sample. To obtain the photostability χ, i.e. the ratio of undamaged strands per irradiation dose of a specific 3-mer sequence, the number reads of all sequences containing this specific 3-mer sequence were summed up before normalization, as done previously for tetramers and hexamers^32^ (see methods and SIAppendix section 2).

Figure 2B displays ratios of undamaged strands χ of a selection of 3-mers sequences obtained in this way as a function of absorbed dose in units of photons per base (PbB). As expected, pyrimidine-rich sequences such as d(TTT) or d(TTC) show the highest and guanine-rich sequences the lowest damage rates, which is in good agreement with case studies of the damage quantum yields of individual DNA photolesions.^24,32^ The damage rates μ shown in Figure 2C of each trimer sequence are then derived from the initial slopes of damage profiles of Figure 2B (see methods). Going beyond previous literature, this data allowed us to calculate the damage rates of each lesion as a function of nearest neighbors, which is essential for accurate modeling of longer strands.

As an example, we addressed the difference in damage rates for d(TTA), μ_*TTA*_ = 28 × 10^–3^1/*PbB*, and d(TTG), μ_*TTG*_ = 20 × 10^–3^1/*PbB*. The type of purine in the vicinity of the TT strongly influences damage formation, directly indicating the context dependence of the photo lesion formation. This finding is supported by related data on tetramers where the masking of a central TT dimer by neighboring guanines resulted in a 5-fold reduction of lesion formation. The data further showed that 84% of all possible 3mer sequences have damage rates below 20 × 10^–3^ damages per photons per base, which indicates a relatively high UV stability of most codons (see Table S1 for a complete listing). This is plausible, as the canonical nucleobases themselves were presumably selected to be UV-resistant.^18^

### Photostability Calculation for Protogenome Pools and Chronologies

Once the pools of 10^4^ different sequences with a predefined, codon-generated sequence bias are generated for each step *M* of a chronology *C*, we exposed them in silico to light using the Monte Carlo approach to calculate the formation of the UV damage (see Methods and SIAppendix sections 4, 5). We exposed each pool to the same fixed dose of two absorbed photons per base (2 PpB), which was chosen to be realistic on the early Earth, where this should correspond to an illumination period of less than 4 min (see SIAppendix section 1). The resulting photodamage is taken as a measure of the UV susceptibility of the protogenome.

The irradiation dose is high enough to cause severe damage with χ = 0.06 in the most UV-susceptible pool (Figure 2D, for 150mers from TTT and AAA). At the same dose, only weak UV damage, χ = 0.89, occurs in pools with comparably UV-stable sequences (Figure 2D, 150mers from GTG and CAC). The strongly differing ratios of undamaged strands χ between both types of pools, even at small doses of 2 PpB, show the importance of selecting a suitable sequence bias for an early life form under UV irradiation.

In the following, we calculate the UV stability in each step *M* of chronologies *C* proposed in the literature, which was obtained based on specific physical, chemical, or combinatorial boundary conditions but omitted the effects of UV irradiation (see Table S2). While some publications propose chronologies of trimer pairs,^9,10^ other works refer to chronologies of amino acids.^37,38^ To keep these different approaches comparable, we converted the latter into chronologies of trimer pairs as detailed in SIAppendix section 3 and displayed all chronologies in Table S2. Many^10^ of the chronologies listed here have already been combined into a so-called consensus chronology, which we also include below for comparison.^10^

**Figure 3:**
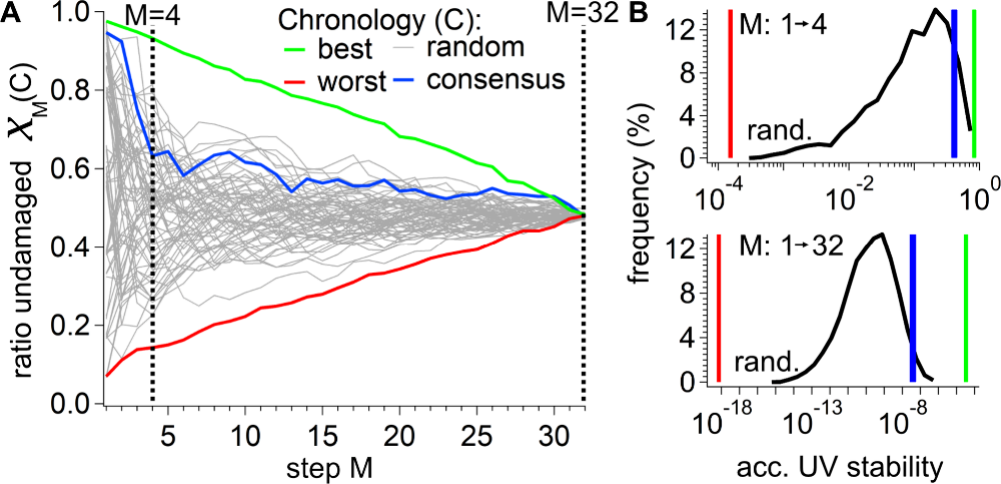
Calculated UV sensitivity of protogenomes over their codon development. (A) The UV susceptibility was calculated for different codon chronologies implemented, as shown in 1C. The fraction of the undamaged strands as a function of the development step M for proto-genomes composed of 150 bases. Marked in gray are codon chronologies in which the codon order was randomly chosen; marked in green and red are the most or least UV-stable codon chronologies found in this study, respectively. Marked in blue is the consensus chronology,^11^ showing a significantly higher UV stability than most random chronologies. (B) Probability distribution of the accumulated UV stability of randomly chosen codon chronologies (black) after four (*M* = 4, top) steps, which corresponds to 8 available amino acids and suffices for the formation of simple proteins, and at the end of the proto-genome development (*M* = 32, bottom). The *x*-axis is scaled logarithmically, as the accumulated UV stability, χ_*acc*,*N*=32_(*C*)=∏_*M* = 1_^*N* = 32^χ_*M*_, is obtained by multiplying the UV stabilities of the individual steps and, therefore, spans many orders of magnitude. The accumulated UV stabilities of the most (green), least (red) UV stable and the consensus^10^ chronology (blue) are marked by vertical lines.

Since the absolute UV stabilities of a chronology depend on the irradiation dose, they must be seen relative to reference chronologies obtained using the same fixed irradiation dose. In Figure 3A and S2, we show examples of the UV stability traces χ_*M*_ (lines in Figure 3A) and compare the consensus chronology (blue trace) with the most UV-stable (green trace) and most UV-susceptible chronology (red trace) obtained from the numerical model (see SIAppendix section 6). Interestingly, in the first step of the chronologies, one typically finds the most extreme values for UV stability. Here, the damaging potential of the two trimers of a single codon and its reverse complement determines the overall result. Upon the addition of another codon, the damaging potential has a less pronounced effect. At the end of the chronology, so many codons are present that the finally added codon only has a negligible effect.

In numbers, the UV stability of the limit chronologies ranges between χ_*M*=1_(*C*_*best*_)=0.98 and χ_*M*=1_(*C*_*worst*_)=0.06 in the first step and converge to χ_*M*=32_ = 0.48 in the last step of their development (see Figure S2 for details). The convergence of the UV stabilities χ_*M*_(*C*) of all chronologies in their final evolutionary step *M* = 32 is expected due to the disappearance of a sequence bias. Here, all protogenomes comprise all possible trimer sequences (32 trimer pairs, 64 trimer sequences) and are thus no longer distinguishable in our model. Table 1 shows the codon appearance order for the most UV-stable and the most UV-susceptible chronologies. Guanine-rich codon pairs yield a high UV stability, and dipyrimidine-rich codon pairs a low UV stability. When single dT and d(CC) sequences are part of UV stable trimers, they are flanked by dG.

**Table 1:** Most UV-Stable (A) and Most UV-Sensitive (B) Codon Chronologies Are Shown As Green and Red Lines in Figures 3, 4, Respectively^a^

*M*	codon		*M*	codon	
1	GCG	CGC	17	GGG	CCC
2	GCA	TGC	18	CTA	TAG
3	ACG	CGT	19	ACT	AGT
4	ACA	TGT	20	TCA	TGA
5	GTG	CAC	21	GAT	ATC
6	GGC	GCC	22	CAA	TTG
7	CCG	CGG	23	GAG	CTC
8	ATG	CAT	24	GGA	TCC
9	GGT	ACC	25	CCT	AGG
10	GAC	GTC	26	GTT	AAC
11	GTA	TAC	27	TCT	AGA
12	GCT	AGC	28	TTA	TAA
13	CCA	TGG	29	ATT	AAT
14	CTG	CAG	30	CTT	AAG
15	TCG	CGA	31	GAA	TTC
16	ATA	TAT	32	TTT	AAA
M	codon		M	codon	
1	TTT	AAA	17	TCG	CGA
2	GAA	TTC	18	GGG	CCC
3	CTT	AAG	19	CCA	TGG
4	TTA	TAA	20	ATA	TAT
5	ATT	AAT	21	GAC	GTC
6	TCT	AGA	22	ACA	TGT
7	CAA	TTG	23	GGT	ACC
8	GTT	AAC	24	CTG	CAG
9	GGA	TCC	25	ATG	CAT
10	CCT	AGG	26	GTA	TAC
11	ACT	AGT	27	GTG	CAC
12	TCA	TGA	28	CCG	CGG
13	GAG	CTC	29	GGC	GCC
14	GAT	ATC	30	ACG	CGT
15	CTA	TAG	31	GCG	CGC
16	GCT	AGC	32	GCA	TGC

a Both chronologies were calculated according to the algorithm described in SIAppendix section 6.

Which would be the most likely UV stability if the chronology of sequence bias were to develop purely by chance without selection pressure? To address this question, we computed the UV stabilities of 10^4^ random development chronologies, in which at each step, *M* = 1,..,32, the newly added trimer pairs were selected purely by chance and show an excerpt of 250 randomly chosen chronologies from this pool as gray lines in Figure 3A. The UV stability of most of these random chronologies ranged approximately halfway between the most UV-stable and the most susceptible chronology. Surprisingly, however, the consensus chronology introduced above (blue line) is well above most gray curves displaying random chronologies.

While the UV stabilities of the random chronologies vary widely in the first few steps of the chronology, they become much more similar after about *M* = 4 as the relative number of sequence patterns with extreme UV-damage values decreases in the further development steps (see Figure S3). Therefore, particularly early proto-life, which only used a few codon sequences and thus few amino acids, would benefit most from an optimal choice of codons.

The accumulated UV stability χ_*acc*_(*C*) provides a suitable metric to compare different chronologies *C* as it aggregates the complete history of UV stabilities of a chronology into a single measure and describes the relative impact of UV light on this chronology. In Figure 3B, we show histograms (black traces) of the early accumulated UV stabilities for chronologies until step 4, χ_*acc*,4_(*C*) (top), as well as of the complete accumulated UV stabilities, χ_*acc*,32_(*C*) (bottom). The distributions show the probability (*y*-axis, black) of obtaining a specific accumulated UV stability within the random development chronologies.

The positions of the accumulated UV stability of the consensus chronology (blue), the most stable (green), and most susceptible (red) UV chronologies are represented by vertical bars. The accumulated UV stabilities range over several orders of magnitude. They are found to be in the range from 1 to 10^–4^ after step 4 and between 10^–4^ and 10^–18^ after the last step. The accumulated UV stabilities shift to lower values when more steps have to be considered. Chronologies with the largest values of χ_*acc*,4_(*C*) or χ_*acc*,32_(*C*) contain G-rich, UV-resistant codons already in the very first steps. The consensus chronology (blue) has a significantly higher accumulated UV stability than the most probable UV stabilities found in random developments protogenomes (peak of the black curve).

**Figure 4:**
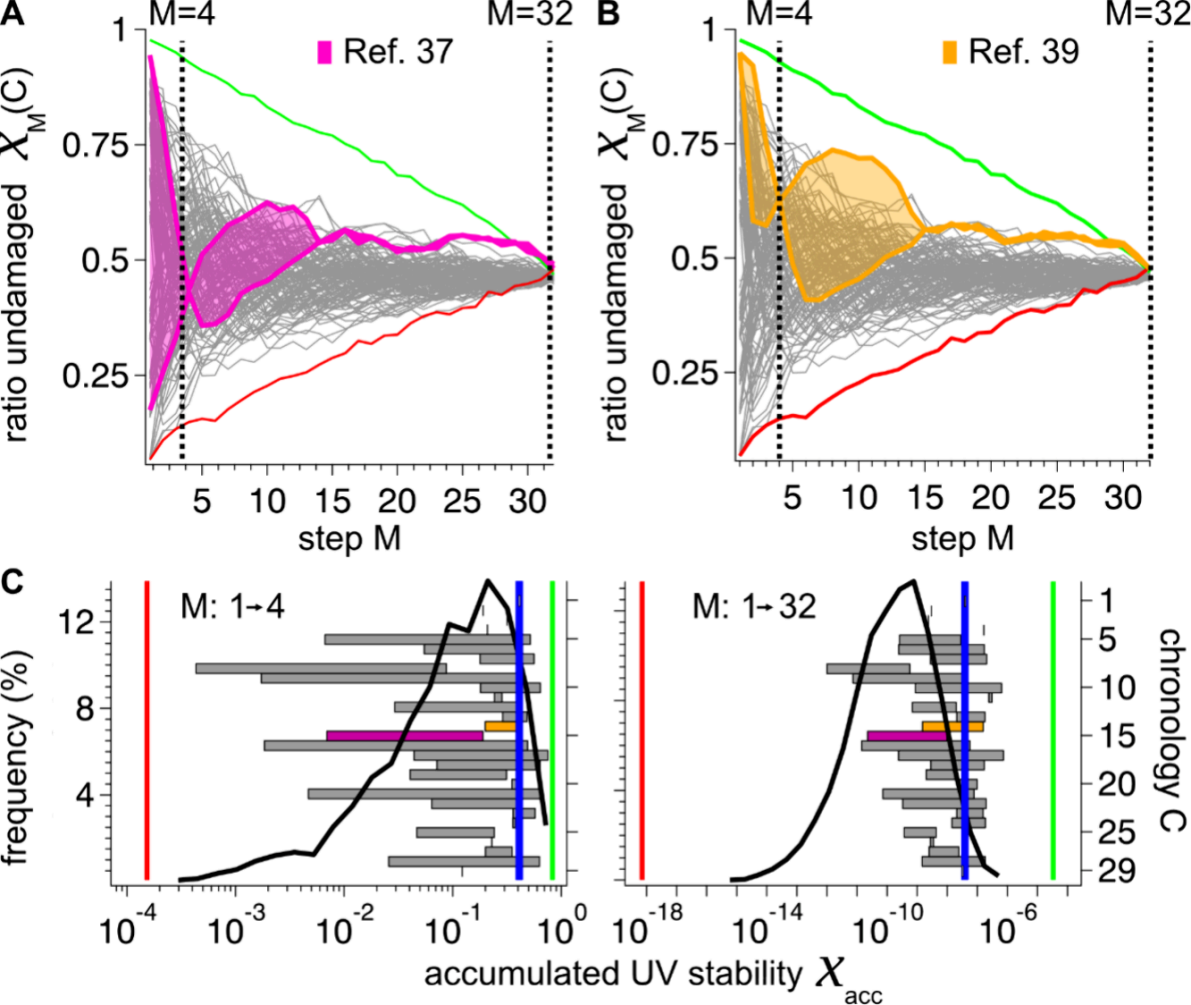
Comparison of the calculated UV stability for literature codon chronologies. Chronologies with randomly chosen codon pairs at each step (gray), best (green), and worst (red) case curves are shown as in Figure 3 and compared to exemplary chronologies 15 (A, purple) and 14 (B, orange), both derived from the references^39^ and.^37^ Both chronologies are ambiguous in specific developmental steps in the choice of the next codon pairs (A: *M* = 1–4, *M* = 4–14, B: *M* = 2–4, *M* = 4–15). The resulting chronologies cover a range of different UV stabilities χ, which are shaded in color. (C) Probability distribution of (left *y*-axes, black curve and vertical lines) of accumulated UV stability χ_*akk*_ as in Figure 3B. Horizontal bars: Range in accumulated UV-stabily for the Chronologies defined in Table S2 (right *y*-axis indicates the criteria index). The ambiguities of codon selection as shown in A (purple) and B (orange) also result in a wide range of χ_*acc*_. Unambiguous chronologies (e.g., C: 1–4) are shown as thin vertical markers. Detailed listings are found in Table S2, Figure S2. At the end of the evolution, most literature chronologies show higher UV-stability than randomly chosen ones.

Since the consensus codon chronology is a compilation of several chronologies based on various codon constraints unrelated to UV stability, the UV stability of the protogenomes associated with these chronologies should be addressed. In Figure 4A, B, we show an example of the UV-stability χ_*M*_(*C*) of two example chronologies^37,39^ (purple and orange) that were included in the consensus chronology and compare them with the randomly generated (gray), as well as the most UV-stable (green) and the most UV-susceptible (red) chronologies. Remarkably, although both chronologies are based on entirely different concepts (protein folding rates, purple vs amino acids on meteorites, orange), they are compatible with high-intensity UV radiation.

In some amino acid chronologies reported in the literature, one of several trimer-pairs can be added interchangeably in specific steps, leading to different sets of codon chronologies for a single amino acid chronology. Figure 4 shows the achievable range of UV stabilities of such chronology sets by color shading (see SIAppendix section 6). If this ambiguity is pronounced during the early steps of a chronology (see Figure 4A vs 4B), a large range of accumulated UV stabilities could have been implemented by the respective chronology (Figure 4C, purple vs orange bar and the gray bars for other published chronologies). For details, see Table S2 and Figure S2. As a common feature, one may extract that the chronologies with the highest UV stability used guanine-rich codons early and flanked the first emerging codons featuring dipyrimidines by G nucleotides.

For all literature chronologies C listed in Table S2 (Figure 4C, right axis), most of which were also included to construct the consensus chronology, we display the accumulated UV stabilities after step four, χ_*acc*,4_(*C*), and after the last step, χ_*acc*,32_(*C*), as horizontal bars (in Figure 4C), that range between a minimum to a maximum accumulated UV stability. At an early development step (*M* = 4, Figure 4C left), the accumulated UV stability of the literature criteria ranges from 10^–4^ to 10^–1^, covering the complete range of fully randomized chronologies. At late stages (*M* = 32, Figure 4C right), the accumulated UV stability of the literature criteria ranges from 10^–13^ to 10^–7^, showing higher accumulated UV stabilities than found in random chronologies.

## Discussion and Summary

Modeling the development and sequence composition of the genome pools available to the first more complex life forms on early Earth is a scientific challenge. Various environmental factors could have selected genomes and the comprising sequence patterns. We focused here on quantifying the UV stability of protogenomes at the early stages of life. Our numerical models are based on previously unavailable sequence-context-dependent UV-damage rates of DNA, which we obtained experimentally by high throughput sequencing.^32^ As expected, we found that guanine-rich sequences exhibit high UV stability. In addition, guanine adjacent to a dipyrimidine such as d(TT) significantly reduces the formation of dimer photolesions. Guanine-containing sequences have also been shown to promote sequence-selective self-repair of pyrimidine dimer lesions, potentially providing a dominant repair pathway before the evolution of enzymatic repair. Using these results, we modeled the UV stability of protogenomes in a general way based on their sequence bias that results from the accessible set of codon sequence patterns and their change during the developmental steps of a hypothetical proto-lifeform.

This allowed us to compare the UV compatibility of different literature models that predict the chronology of the development of codons and amino acids and characterize the UV-induced sequence bias that would occur in the respective genomes. We have found that most of these models generate UV-stable protogenomes through the early use of guanine and cytidine-rich codons and the effective flanking of pyrimidine dimer sequences by guanine nucleotides. This observation supplements the inherent preference for guanine- and cytosine-rich polymers in the Origin of Life context: The preference for GC-rich sequences coincides with the efficient polymerization of 2′,3′-cGMP, whose product serves as an early template for polymerizing other nucleotides.^28^ In addition, GC-rich RNA polymers have a high melting temperature, which efficiently protects them from hydrolysis by preferring their double-stranded form.^40^ The findings are interesting as the respective protogenomes are particularly resistant to UV radiation. Indeed, a comparison with the literature chronologies shows that UV compatibility can also be achieved by assuming other chemical, physical, or combinatorial boundary conditions.

The metric of accumulated UV stability introduced here provides an effective tool to compare the different chronologies and their developing sequence biases discussed above. While the low accumulated UV stability of a particular chronology would indicate a rapid accumulation of UV damage on the respective protogenomes and thus their probable extinction, protogenomes following chronologies with a high accumulated UV stability were more likely to survive the solar radiation conditions on the surface of the early Earth.

We found that early protogenomes benefit most from choosing an optimal UV-resistant set of codon sequences. Since early life forms likely only had access to a limited set of amino acids and associated codon sequences at an early stage, a development along a chronology with high accumulated UV stability would, therefore, be essential under UV irradiation. Previous studies have shown that 8–10 different amino acids are mainly sufficient to form functional proteins.^41^ Accordingly, our data show that the UV stability of protogenomes at up to 4–5 developmental steps, i.e., with access to 8–10 different codon sequences and the corresponding amino acids, is massively influenced by the choice of codon sequences used. In subsequent developmental stages, the selective effect of UV irradiation on protogenomes rapidly decreases, suggesting that higher evolved life forms were less constrained by UV light in their choice of subsequent codon sequences.

In this paper, we have characterized the potential influence of UV irradiation on the development of genomes in protogenomes using a DNA model system. Although the exact timing of the first use of DNA in early life is unclear, our model presents a proof of principle on how sequence-dependent damage rates can be used to infer the chronology of codon evolution, which can be readily extended to RNA systems once sequence-dependent damage data are available. Here, the concentration on single-stranded DNA, only present for a short time during the replication reactions, offers a worst-case model, since the chemical longevity of ssDNA in combination with the increased UV absorption compared to double-stranded DNA would lead to a particularly high accumulation of photodamage.

Within the scope of this model, we found that codon chronologies based on UV compatibility are surprisingly compatible with published chronologies that do not consider UV damage. The results further allow us to specify the UV-induced codon selection in ambiguous steps of published chronologies (see Figure 4C). The presented analysis can be transferred to extraterrestrial environments and thus has the potential to predict the development of a codon pool on exoplanets under exposure to UV irradiation in case we would find evidence for an RNA-DNA-based life there. UV light could have been a dominant selection criterion in a meteoritic setting. Our approach lays the ground for future studies on radiation effects on other nucleotide systems. It also supports the idea that if protogenomes have developed on the surface of the early Earth, UV-induced selection processes had likely played a crucial role in the early stages of life when the genetic code was developed.

## Materials and Methods

### Determination of Trimer Damage Rates

The data set used to determine the neighbor-dependent damage rates from trimer DNA is also published in the NCBI Sequence Read Archive (SRA), accession number PRJNA929909 (https://www.ncbi.nlm.nih.gov/sra/PRJNA929909) and was obtained as described before by irradiating a synthetic 16mer ssDNA strand ACACNNNNNNNNACAC comprising 8 randomly chosen, canonic nucleotides (N) at the center with specific dosages *D* displayed in table S3. The^32^ irradiated samples were prepared for Illumina sequencing using the standard protocol of the Swift Accel-NGS 1S DNA library kit (Swift Bioscience, USA). The used polymerases in this kit stall reliably upon reaching dimeric photolesions, leading to fewer reads for sequences more prone to UV damage. A sequence-by-sequence comparison of read counts between the irradiated and nonirradiated control samples yields the normalized ratio of undamaged strands *S*(*j*,*D*) for each full-length sequence *j* (see SI-e4) of all possible 4^8^=65536 distinct randomers. As described in detail in Section 2.2 of the SI Appendix, the molecular damage rates given in Table S4 were determined by focusing on the linear drop of the ratio of undamaged sequences in the low-dose regime up to two absorbed photons per base. This ensures minimal crosstalk between different photodamages on one strand and allows to deconvolute measured damage rates of the full randomer sequences into the molecular (dimer, Table S4), trimer (Table S1) and tetramer (Table S5) damage rates (see equation SI-e5). Comparison of the tetramer and trimer damage rates with the molecular damage rates yields the impact of neighboring bases for each possible dimer as described in SIAppendix section 2.5.

#### In Silico Irradiation Using a Monte Carlo Approach

Using the neighbor-dependent damage rates obtained in this way for all possible dimer sequences, the damage on exposed DNA strand with any sequence can now be determined. For each step of a chronology C (defined in Table S2), we first create a pool of 10^4^ 150mers, which are randomly assembled from the codon sequences available for this step (see Figure 1C and SI Appendix). Photons are then distributed over all strands of this pool in accordance with the fixed dose of 2 absorbed photons per base based on a Poisson distribution (i.e., a total of  per pool). The absorption per base was assumed to be approximately equal^42^ so that no adjustment of the photon distribution across the strand is necessary, as verified in SIAppendix 5 and Figure S4. For each strand of the pool, the absorbed photons are then converted into photodamages for each contained dimer according to the measured context-dependent dimer damage rates (see SIAppendix section 2.5). The number of strands without a single damage is then normalized by the total number of strands in the pool, resulting in the ratio of undamaged strands χ as shown in Figures 3 and 4. This calculation is performed independently for each step of a chronology. This means that the respective strands and damage states of step M are not used in step M+1, but that the sequence bias of the pool in step M+1 differs only by the contribution of an additionally accessible codon pair. This model is plausible since it can be assumed that the development leading to the addition of new amino acids and, thus, codon sequences in the genome of a proto-life form spans over many replication cycles. With a uniform irradiation intensity, strands from pools with a small χ that are particularly susceptible to UV damage would likely not be able to be replicated as well as UV-resistant strands from pools with a large χ. Distributions of accumulated UV stabilities χ_*acc*,*M*_ as shown in Figure 3B and 4C are displayed as histograms that collate the UV stabilities χ_*M*_ within x-intervals of a constant width (factor 1.5) in the logarithmic scale.

## Supporting Information Available

Estimates of the absorbed UV dose on the early Earth, explanations of the calculation of the dimer and trimer damage rates, displayed in Table S1, and the approach used to analyze the sequencing data. Furthermore, the method for deriving the codon chronologies from amino acid sequences is explained, and the Monte Carlo method is used to calculate the UV susceptibilities of the protogenomes, taking into account the nearest neighbor effects. Supplementary Figures show an error estimation of the numerical method, by the combinatorial occurrence of dimers, and display the chronologies of UV susceptibility of all amino acid/codon sequences listed in Supplementary Table S2 (PDF)
SequenceData_PositionCorrelation (ZIP)
Transparent Peer Review report available (PDF)
