# Supplementary material for: Selection of Early Life Codons by Ultraviolet Light
Source: ACS Cent Sci. 2025 Jan 8;11(1):147–56. doi: 10.1021/acscentsci.4c01623 (PMC11758376; doi:10.1021/acscentsci.4c01623)
Supplement: Supplementary file 1 — oc4c01623_si_001.pdf [file oc4c01623_si_001.pdf]

Supporting Information for
Selection of Early Life Codons by Ultraviolet Light.

Corinna L. Kufner[1], Stefan Krebs[2], Marlis Fischaleck[2], Julia Philippou-Massier[2], Helmut Blum[2], Dominik B. Bucher[3], Dieter Braun[4], Wolfgang Zinth[5] and Christof B. Mast[4,*].

[1]Harvard-Smithsonian Center for Astrophysics, Department of Astronomy, Harvard University, 60 Garden Street, Cambridge, MA 02138 (USA)

[2]Laboratory for Functional Genome Analysis, Gene Center, Ludwig-Maximilians-University Munich, Feodor-Lynen-Straße 25, 81377 Munich, Germany

[3]Department of Chemistry, Technical University of Munich, Lichtenbergstr. 4, 85748 Garching b. München

[4]Systems Biophysics, Ludwig-Maximilians-University Munich, Amalienstr. 54, 80799 Munich, Germany

[5]BioMolecular Optics and Center for Integrated Protein Science, Ludwig-Maximilians-University Munich, Öttingenstrasse 67, 80538 Munich, Germany

*Christof B. Mast.
Email: christof.mast@physik.uni-muenchen.de

This PDF file includes:

Supporting text
Figures S1 to S4
Tables S1 to S5
SI References

Other supporting materials for this manuscript include the following:

Software S1

Supporting Text

1. Approximation of absorbed dose in an early life context.

To approximate the magnitude of UV radiation absorbed by DNA in the context of an early Earth, we use Lambert-Beer's law. We deduce the total number of photon absorptions per time:

(SI-e 1) $N = \int I(\nu) \frac{1}{h\nu} \epsilon(\nu) \cdot \ln(10) \cdot \frac{1}{N_A \cdot 10^{-3}} d\nu$

with the intensity spectrum $I(\nu)$ of an early Earth[1] (zenith angle: 0°, albedo: ocean), the photon frequency ν, the Planck constant h, the extinction coefficient $\epsilon(\nu)$ of thymine[2], and the Avogadro constant N_A. We assume that the nucleotides are located on the surface, e.g. in the uppermost millimeters of a pond, so that no further assumptions regarding the nucleotide concentration or the nature of the water with regard to the absorption of UV light are necessary, which would significantly affect the general validity of the considerations of this study. We perform the integral between 250 and 280 nm and yield $N = 35\, photons/hour$, corresponding to 2 photons per base in 3.5 minutes.

2. Determination of damage rates for single-stranded DNA trimers

2.1. Illumination and sequencing of damaged DNA strands. To model UV damage of long strands of arbitrary sequence, it was necessary first to measure the formation of the most abundant dimeric photo lesions per adsorbed photon (damage rate) under well-defined experimental conditions for all permutations of neighboring bases. While the formation of dimeric lesions has been studied in detail under different conditions (e.g., cyclobutane pyrimidine dimers as a function of the adjacent bases)[3–7], a uniform set of dimeric damage rates, including adenine-adenine photoproducts as a function of all neighboring bases under uniform conditions, has not been available so far.

However, the determination of the individual damage rates for each of the dominant dimer lesions of two pyrimidine bases (TT, CT, TC, CC) or two adenosine bases (AA), and for each possible neighbor sequence would require 4^4 (2 neighbors on each side) x 10 (dimer combinations without order) x 12 (typically required single dose values) = 30720 singular irradiation experiments and downstream HPLC runs. To avoid this, we chose to irradiate single pools of randomized, short DNA strands instead with subsequent analysis through high throughput sequencing[8].

We prepared the samples from lyophilized commercially synthesized single-stranded DNA (ssDNA) with a length of 16 nucleotides and sequence 5'-ACACNNNNNNNNACAC-3' (biomers, Germany) by dissolution in spectroscopically pure water (LC-MS grade, Carl-Roth) and titration to 1x PBS buffer conditions (137 mM NaCl, 2.7 mM KCl, 10mM Na_2HPO_4, 1.8 mM KH_2PO_4) to maintain a pH of 7.4 and a DNA base concentration of 1 mM. The samples were centrifuged and vortexed, and the final concentration was checked by Nanodrop (Thermofischer, US) and UV-Vis measurement (Shimadzu UV1800). The DNA sequences consist of three parts: a central part containing eight random canonical nucleotides and two flanking parts of the sequence d(ACAC), which act as recognition tags in sequencing analysis.

3.45 ml of buffered DNA solution was irradiated in a fused silica cuvette (type 117100F-10-40, Hellma, Germany) with a path length of 10 mm. During irradiation, the cuvette was kept at a constant temperature of 22 °C and mixed by magnetic stirring. The sample was irradiated by a Nd-based laser system (AOT-YVO-25QSP/NOPA, AOT, UK) with a repetition rate of 6.5 kHz, an average power of 20 mW, at a wavelength of 266 nm. The laser power was checked before and after passing through the sample using beam splitters and appropriately placed power meters (Ophir, Israel). The difference between the two readings was used to determine the total absorbed dose in each case (see Table S3 and SI appendix 2.4). After the desired dose was reached, exposure was interrupted for a short time to collect 50 µl of the sample, which was then frozen at -80 °C for subsequent high-throughput sequencing. Twelve samples in total were

collected in duplicate with exponentially increasing doses from 2 to 560 photons per base. The residual non-irradiated DNA solution was kept as a control.
All samples were prepared for sequencing according to the standard protocol of the Swift Accel-NGS 1S DNA library kit (Swift Bioscience, USA). Special care was taken to process the samples immediately after thawing to avoid thermal alteration of the damage state of the strands. Sequencing was performed using a Hi-Seq high-throughput sequencer (Illumina, USA) with a planned number of 40 to 60 million reads per sample.

2.2. Analysis of sequencing data. The principle for determining context-dependent damage rates of sequences is based on the inherent property of the high-fidelity polymerases used for sequence library generation to stall the polymerization process when noncanonical bases are present[9]. Using a complete random sequence pool, that is, every possible sequence of the central randomized part of the DNA occurs at least once, the library preparation procedure leads to a detectable decrease in successful sequencing reads for sequences containing UV lesions. Since we have assigned the amount of sequencing reads to be 40 to 60 million and $4^8 = 65536$ different sequences are possible for a sequence with 8-random nucleotides, each possible sequence will ideally occur about 1000 times in our unexposed sample. Accordingly, the dynamic range for UV damage detection will span approximately three orders of magnitude, enabling us to identify the most common UV lesions that occur more frequently than 10^{-3} occurrences per strand in a complete sequence context. Due to sequence bias in synthesis and sequencing, we expect significant variations for each sequence, which are identical for all samples used since they were obtained from the same stock solution.
As described in[8], the only difference between the unexposed control and the exposed samples is the reduced amount of reads due to the stalling effect of the polymerase used on the damaged sequences. The inherent sequence bias from synthesis and sequencing can therefore be eliminated by normalizing the amount of reads, $Q_j(D)$, per 16-mer sequence j for a sample that was exposed to the dose D with the amount of reads $Q_j(0)$ of the non-exposed control sample to obtain the survival probability. In the simplest case, such as for low exposure doses, one can assume that the decrease in the amount of undamaged strands dQ_j is proportional to their current number Q_j, the differential dose dD and the global damage rate μ_j which translates to the linear differential equation:

(SI-e 2) $\frac{dQ_j}{dD} = -\mu_j \cdot Q_j$

With the monoexponential solution:

(SI-e 3) $Q_j(D) = Q_j(0) \cdot \exp(-\mu_j \cdot D)$

For higher dosages, other damage states and back reactions must be considered. Mainly, if the sequence *j* contains a more significant number of di-pyrimidines, a bi-exponential model applies (see Fig 2B for, e.g., TTT trimers)[8]. In the calculations below, the former case with low dose values in the range of 2 photons per base applies, which is why it is sufficient to use the initial slope of the simple exponential model SI-e3 as the damage rate.
Since we are not only interested in the damage rates of the entire strand but also all subsequences, j can be chosen as a shorter sequence in a central position. In this case, the number of undamaged strands containing a central subsequence j after a dose $Q_j(D)$ is then obtained simply by summing up all sequence reads that contain the subsequence *j* at the center position. In addition, the part of the damage that is not formed within sequence *j* must then be excluded. We achieve this by a normalization step for a non-damaging sequence, j_G = poly-G, of the same length as *j*.

(SI-e 4) $S(j,D) = \left[\frac{Q_j(D)}{Q_j(0)}\right] / \left[\frac{Q_{j_G}(D)}{Q_{j_G}(0)}\right]$,

$Q_{j_G}(D)/Q_{jG}(0)$ is the number of surviving strands determined from sequencing with a poly-G sequence at the central position known to be negligibly damaged at doses up to 500 photons per base[8]. The corresponding data for subsequences of length 3 (trimers) is shown in Fig. 2B (dots), including the bi-exponential fits (lines).
Irrespective of j being a subsequence or the entire sequence of the strand, the molecular damage rates of the respective dimer damage can now be calculated from the damage rates. For this purpose, we split the damage rates using the example of the monoexponential damage model SI-e 3, here for a trimer subsequence $j = XYZ$ with XYZ being a specific sequence made from three canonical bases:

(SI-e 5) $\mu_j = \Delta\mu_X + \Phi_{XY} + \Phi_{YZ} + \Delta\mu_Z$

where $\Delta\mu_X$ and $\Delta\mu_Z$ denote contributions from possible lesion formations with the adjacent start and end sequences[8]. In our case of considering sub-sequences, e.g. trimers within the 16mers strands, the interaction with the neighboring sequences results from all possible molecular damage possibilities with the nearest adjacent base:

(SI-e 6) $\Delta\mu_X = 0.25 \cdot (\Phi_{AX} + \Phi_{CX} + \Phi_{GX} + \Phi_{TX})$,
$\Delta\mu_Z = 0.25 \cdot (\Phi_{ZA} + \Phi_{ZC} + \Phi_{ZG} + \Phi_{ZT})$

The global damage rates μ_j are now determined by the fit function SI-e 3. This procedure is valid for the low-dose case used in this work (D < 2 PpB), allowing for virtually independent damage events. For all possible sequences $j = XYZ$, SI-e 5 defines a system of linear equations, which can be written in general terms as

(SI-e 7) $\mu_j = \sum_{ik} A_{ik} \cdot \Phi_k$

With k being all possible dimer sequences and $A_{i,k}$ a coupling-matrix derived from SI-e 5 and SI-e 6. The solution of this equation system yields the molecular dimeric damage rates Φ_k presented in the SI appendix table S4. To determine the error of the molecular damage rates $\Delta\Phi_k$, the SI-e 7 system was solved for 1000 different μ_j' values, which are sampled with a Gaussian distribution with the width of the fit-error around the fitted rate μ_j. The width of the resulting Gaussian distribution of solutions Φ_k' then defines the error $\Delta\Phi_k$.

2.3. Dimeric and trimeric damage rates. Using the procedure described in the section above, we were able to determine the molecular damage rates Φ_j for all dimers j and damage rates μ_j for all possible trimers j. The detection of the specific damage type was of secondary importance here since it is assumed that the dimer damage considered can at least affect the readout processes in proto-genomes, similar to impairing the function of modern polymerases. Thus, we obtain the following values for the effective damage rates of the dimers from solving SI-e 7. The normalized damage rates μ_j of the trimers shown in Fig. 2C and displayed in Table S1 are determined using the fitting procedure with the monoexponential model SI-e 3.

2.4. Approximation of adsorbed dose. To determine the number of actually absorbed photons N_{abs}, the total number of bases N_{base} and thus the actual dose of the respective samples, the power difference W_{abs} of the laser beam in front of and behind the sample is determined over the entire measurement period. Here, reflections at the cuvette are considered by prior calibration. The number of absorbed photons is, thus, $N_{abs} = \int W_{abs} dt/h\nu$.
The actual number of bases is determined from the strand concentration of the 16mers after sample preparation by a dedicated UV absorbance measurement. Due to the sequence bias introduced by the alternating surrounding start and end sequences (ACAC), the extinction coefficient ϵ_{16} is determined by piecewise averaging over all pairwise base combinations [2] $\epsilon_{i,j}$, $i,j \in \{A,C,G,T\}$:

(SI-e 8) $\epsilon_{16} = \frac{\sum_{i=1}^{15} \varepsilon_{i,i+1} - \sum_{i=2}^{15} \varepsilon_i}{16} = \frac{1}{16} \cdot$
$(\epsilon_{A,C} + \epsilon_{C,A} + \epsilon_{A,C} + \epsilon_{C,N} + 7\epsilon_{N,N} + \epsilon_{N,A} + \epsilon_{A,C} + \epsilon_{C,A} + \epsilon_{A,C} - 3\epsilon_A - 3\epsilon_C - 8\epsilon_N)$

With $\epsilon_N = \frac{\varepsilon_A+\varepsilon_C+\varepsilon_G+\varepsilon_T}{4}$, $\varepsilon_{C,N} = \frac{\varepsilon_{C,A}+\varepsilon_{C,C}+\varepsilon_{C,G}+\varepsilon_{C,T}}{4}$, $\varepsilon_{N,A} = \frac{\varepsilon_{A,A}+\varepsilon_{C,A}+\varepsilon_{G,A}+\varepsilon_{T,A}}{4}$ and $\varepsilon_{N,N} = 1/16 \cdot \sum \epsilon_{i,j}$.
The total number of bases N_{base} is then calculated using the Lambert-Beer Law: $N_{base} = V \cdot N_A \cdot \frac{A_{260}}{\epsilon_{16} \cdot d}$ from the absorbance A_{260} at the wavelength $\lambda = 260nm$ in the sample cell with a path length d in a separate experiment.

2.5 Neighbor-dependent attenuation of the damage rates. The molecular damage rates determined above can be modified depending on their sequence context. We illustrate this effect using the example of the so-called GPy effect, in which the formation of di-pyrimidine lesions is attenuated by a neighboring Guanine[10,11]. One possible explanation is the formation of a charge-transfer state between the strong electron donor G and a pyrimidine, which suppresses damage formation[12,13]. To quantify the effect for the numerical calculations performed above, a set of tetramer damage rates is determined analogously to the trimer damage rates.
Here, one considers only the tetramers that have a di-pyrimidine at the central position. For each di-pyrimidine there are neighboring sequences that, according to Table S4, do not lead to the formation of dimeric lesions within the error threshold of the method used. Examples are TA, CA or AC, AT as their order is not taken into account. As the damage rates in Table S4 have already been corrected for the edge effects, one can directly compare, e.g., the tetramer ATTA, where the TT damage is formed without further interaction with the neighboring bases, directly with GTTA or ATTG to obtain the effect of a single Guanine as the neighboring base. The data in Table S5 reveals an attenuation of the TT damage rate by a factor of 0.7 from one neighboring Guanine ($14{\cdot}10^{-3}/21{\cdot}10^{-3}{\sim}0.7$) and 0.2 for two neighboring Guanines ($4{\cdot}10^{-3}/21{\cdot}10^{-3}{\sim}0.2$). A similar trend is obtained for the CT or CC damage shown in Table S5. By this method, a matrix $T_{W,Z}(XY)$ can now be defined, containing the attenuation factors for the damage of a dimer XY with its neighbors W and Z (WXYZ) and allowing the estimation of UV damage for large, biased sequence pools of long DNA strands.

3. Deduction of codon chronologies from amino acid chronologies.
Often in the literature, not codons but amino acid chronologies are given, i.e., the sequence of amino acids is shown according to their predicted occurrence in evolution[14,15]. When the canonical genetic code existed in this early phase of evolution, these amino acid chronologies provide a plausible starting point for the sequence bias of the proto-genomes, i.e., the genomes of the earliest life forms of that time. However, it cannot be ruled out that other codes were used for the restricted set of amino acids at an early stage. The derivation of codon chronologies from amino acid sequences given here is thus an approximation for the evolution of sequence bias for protogenomes.
For the construction of codon chronologies O_C:c_1,...,c_{32} from amino acid chronologies O_A:a_1,...,a_{21}, an analogous procedure was used to determine the consensus order for codons [14,15]. For each amino acid a of O_A, we:

1. Determine all codons for the amino acid a and their reverse complementary sequences according to the canonical genetic code.

2. Choose from these codon pairs the pair that can be obtained from the already used codon pairs with the change of only one base.

3. If there are several corresponding codon pairs with this property, we choose the codon pair from this group with the highest thermal stability, i.e. the highest melting temperature of the codon-anticodon duplex.

This procedure is repeated until all 32 codon pairs have been arranged. A possible late rearrangement, e.g., by codon capture, is not considered. This approximation is plausible as the selective effect of UV radiation is expected to play a role preferentially for less evolved life forms that have only a limited set of amino acids available.
The chronologies (C) used in Fig. 4C are explicitly shown in Table S2 with their corresponding amino acid sequence. By bracketing the amino acids a_v,..,a_w, we indicate when each of these amino acids can be added interchangeably in a particular step: O_A:a_1,...,(a_v,..,a_w),..a_{21}. This ambiguity of an amino-acid chronology leads to several possible codon chronologies, of which we indicate the most UV-sensitive (red) and the most UV-stable (green) in Figures 3, 4, S2 and S4. In Table S2, these chronologies are labelled as "min" and "max" in the "UV-sensitivity" row.

4. Numerical damage model for random DNA pools.

This work focuses on the UV damage susceptibility of sequence pools that develop from a strong sequence bias towards a balanced sequence space. Since there is an unmanageably large number of different development paths, we randomly select 10000 codon chronologies as introduced in the main text and Figure 1C. This results in a distribution of damage susceptibilities, allowing us to determine how likely the UV susceptibility of a particular amino acid chronology (e.g., from the literature) would occur without any selection pressure.
For this, we use the results from the previous chapters, which yield both the molecular dimer damage rates and the effective damage rates of all other subsequences j with lengths 3 to 8 under uniform boundary conditions. Especially for subsequences of lengths 4 and 6, the influence of the adjacent bases on the formation of the central dimeric lesion is considered to determine the damage formation in large pools of random long sequences, as detailed before in SI appendix section 2.5.

We implemented this simulation in a custom-made LabVIEW program following a Monte Carlo approach (source files included), which consists of several steps:

1. Creation of the sequence pool:
   For the data shown in the main text figures 2D, 3, and 4, we created for each step of a codon chronology a pool of $m_{total} = 10^4$ strands, each consisting of $n = 150$ nucleotides. The strands of the sequence pool associated with the first step (M=1) of a codon chronology C are composed of only one specific trimer sequence and its complementary sequence. The strands of the sequence pool associated with the second step (M=2) of a codon chronology are composed of the previously used trimer pair and another trimer pair (see Figure 1C). For each codon chronology, pools with an ever-larger accessible sequence space are thus created step by step until all 32 possible trimer pairs can occur in the 32nd step (see Fig. 3). The individual codon chronologies differ only in the choice of specific trimer sequences that are used to build up the 150mers in the respective pools randomly. For the simulation, the pool state P is stored by keeping track of all strands, including their sequence, damage state, and the number of photons absorbed for each of the bases they contain.

2. Irradiation-step of a pool in state P with dose D (photons/base):
   First, the photons corresponding to dose D are distributed over all bases of each strand using a Poisson distribution and are stored in P, which yields the photon number $N_{i,s,P}$ at each base position i (starting with $i = 1$ at the 5' end of the strand) of the strand s in P. The absorptivity is assumed to be averaged over all bases. In SI appendix section 5, we compare this approach with a base-specific absorbance and find only negligible differences.

   Since only dimeric UV lesions are considered in this work, for each dimer $XY_{s,\,i}$ occurring in strand s at position i, we:
   a. check if the dimer $XY_{s,\,i}$ is already damaged. If yes, we directly go to step e.

b. determine the number of photons relevant for the formation of a lesion L, $N_{L,s,i,P}$. Bases at the edge of the strand are considered only once, so that for $i=1$: $N_{L,1,s,P} = N_{1,s,P} + 0.5 \cdot N_{2,s,P}$, for $i = n-1$: $N_{L,n-1,s,P} = 0.5 \cdot N_{n-1,s,P} + N_{n,s,P}$ and for all other dimers $N_{L,i,s,P} = 0.5 \cdot N_{i,s,P} + 0.5 \cdot N_{i+1,s,P}$.

c. determine the contextual bases left (W) and right (Z) from the actual dimer at position i and look up the context-dependent damage rate using the attenuation matrix defined in SI 2.5 to obtain the damage probability $p_{dmg} = T_{W,Z}(XY) \cdot \Phi_{XY}$.

d. Execute an unbiased random generator resulting in a real value α between 0 and 1, and decrease $N_{L,s,i,P}$ by 1. $\alpha < p_{dmg}$ confirms the damage formation, saves the change in P and the algorithm directly proceeds with step e. In case of $\alpha \geq p_{dmg}$, step d is repeated until $NL_{s,i,P} = 0$.

e. Go to the next position $i' = i + 1$ if $i < n - 1$, otherwise continue.

3. After all photons for each strand inside P were evaluated and the damage states were updated in P, strands that contain at least one damage are considered to be "dying" strands, while strands without any damage are defined to be "surviving" strands occurring $m_{surv,P}$ times. The value "ratio undamaged" shown in Fig. 3 and 4 accordingly is the defined as the ratio of $\chi = m_{surv,P}/m_{total}$, with m_{total} the initial amount of strands.

The threshold to distinguish surviving and dying strands was defined as 1 because each UV lesion is assumed to be a detriment to the transfer of genetic information to the successor generation. Thus, the quantity χ reflects how many strands emerge from UV exposure without any evolutionary disadvantage. Even if the strands would suffer such a disadvantage only after several damages, this would be compensated by a slightly longer exposure, i.e., a higher dose. Regardless, for a constant dose, the relative susceptibility of these pools to UV irradiation can be read from the comparison of χ for different pools P which exhibit different codon sequence bias.

5. Rationale for using sequence-averaged absorption coefficients in the Monte-Carlo simulations. In the Monte Carlo simulation described above, we use a Poisson distribution of photons across each DNA strand and assume approximately uniform absorption across all bases A, G, C, and T. To confirm that this approximation is sufficient, in the following, we perform and compare the calculation of the fraction of intact strands of the same codon chronologies using the above Monte Carlo simulation once taking into account the base-dependent absorption and once with an averaged absorption option over all bases. For the first case, we modify the simulation in that we change the used dose of N photons per base by a weighting factor α_b for each species b of all bases A, G, C, and T, resulting in a specific dose $N_b = \alpha_b \cdot N$. To determine α_b, we first account for the total absorbed energy E_b per base species b:

(SI-e 9) $$E_b = \frac{hc}{\lambda} \cdot N_b \cdot c_b \cdot \Delta d \cdot A = I \cdot c_b \cdot \epsilon_b \cdot \Delta d$$

with $\frac{hc}{\lambda}$ denoting the energy per photon, c_b the total concentration of base $b \in \{A,C,G,T\}$, Δd and A the thickness and area of the irradiated volume, I the irradiation intensity and ϵ_b the extinction coefficient of each base[8]. From this we obtain:

(SI-e 10) $$\frac{N_b}{\epsilon_b} = \frac{I \cdot \lambda}{h \cdot c \cdot A}$$

From the definition of the average number of absorbed photons $N = \sum_b \frac{N_b c_b}{\bar{c}}$ with the average concentration of bases $\bar{c} = \frac{1}{4} \sum_b c_b$, we directly obtain a relationship between N and N_b:

(SI-e 11) $$N_b = N \cdot \frac{\epsilon_b}{\sum_{b'} \frac{c_{b'} \cdot \epsilon_{b'}}{c}}$$

With $\alpha_b \equiv \frac{\epsilon_b}{\sum_{b'} \frac{c_{b'} \cdot \epsilon_{b'}}{c}}$ as the weighting factor for the absorbed number of photons N. We therefore run the simulation for the codon chronologies given by the criteria 1-8 defined in Table S2, starting with the same number of photons per base $N = 2$ once without any modification (Figure S4a) and once by weighting the poison distribution with the prefactor α_b for each base b separately (Figure S4b). As expected, the resulting change is negligible compared to sequence dependence in the sequence pools and therefore justifies the use of averaged extinction coefficients for the benefit of faster speed of numerical calculations.

6. Evolutionary algorithm for calculation of codon chronologies with worst and best possible UV resistance.

The procedure shown in Fig. 1C applies to well-defined codon chronologies, i.e., when the order of all codon pairs is fixed. To test the UV sensitivity of such a chronology, 32 test pools, each containing 10000 strands of 150 bases in length, are exposed in silico to a fixed radiation dose D following the procedure described above. After irradiation, the number of strands without a single damage is determined for each pool. This number in relation to the number of strands initially present yields the value χ "ratio undamaged". The strands of the first of these pools are randomly formed from the first trimer pair of the codon chronology, the strands of the second pool from the first and second trimer pairs of the codon chronology, and so on. The strands of the last test pool are formed randomly from all available codons and thus have no sequence bias. The first pool has a maximal sequence bias because its strands are formed from only one trimer sequence and its complementary sequence. Each codon chronology is thus a possible evolution from a strongly biased to a completely random sequence pool.

Whereas, e.g., the randomly chosen codon chronologies (gray curves in Fig. 3A and Figs. 4A,B) are unambiguous by definition, for codon chronologies found by external criteria, often no unique sequence can be specified. For example, taking the temperature stability of codon pairs as a selection criterion and defining the codon chronology by sorting them by their melting temperature, some codon pairs, such as GUA/UAC and CUA/UAG cannot be uniquely sorted due to their identical melting temperature.

In a chronology $O_c{:}c_1,c_2,..,(c_v,..,c_w),..,c_{32}$ of codon pairs c_M, this is indicated by writing ambiguously arrangeable pairs $c_v,..,c_w$ in a bracket. To be able to characterize the UV sensitivity for such a codon chronology, it makes sense to determine the two extremal chronologies $O_{c,ext}{:}c_1,c_2,..,c_k,..,c_l,..c_{32}$; $c_k,..,c_l \in \{c_v,..,c_w\}$, for which the codon pairs are sorted either to have a maximal or minimal UV sensitivity.

For this purpose, the uniquely ordered codon pairs of a chronology are first processed as described above, i.e., for each step M, a sequence pool is created whose strands are randomly composed of the codon pairs $c_1,..,c_{M-1},c_M$. This is carried out until in a step $M + 1$, the next codon pair c_{M+1} belongs to a group of interchangeably applicable amino acids $(c_v,..,c_w)$. In this case, we will perform $w - v$ in-silico exposures of a pool of 150meres consisting of the codons $c_1,..,c_{M+1}$, in each exposure choosing a different codon pair for $c_{M+1} \in \{c_v,..,c_w\}$. Finally, the pool with the highest or lowest number of surviving strands can be determined. For the next step $M + 2$, the same procedure is repeated for a codon group $(c_v,..,c_w)$ from which c_{M+1} has been removed. This evolutionary algorithm finally yields the two extremal codon chronologies introduced above $O_{c,ext}$. If several groups of non-arrangeable codon pairs are present, they are processed one after the other in an analogue manner.

A special case will be treated here in particular, namely when there is no external criterion and, thus, all codon pairs can occur in any order. In this case, the above procedure results in the chronologies representing the optimal or worst development of the sequence bias in prebiotic pools purely under UV radiation as the only selection pressure, also shown in Table 1.

Fig. S1. Estimation of the error in determining the proportion of undamaged strands as a function of the number of calculated strands per pool. Exposure settings are the same as for the data shown in the main text Figures 3 and 4: An irradiation dose of 2 PpB, a strand length of 150 nucleotides comprised of codon sequences defined in Table S2, $C=1^{15}$ at step $M = 1$ (a), and step $M = 32$ (b). Each data point corresponds to three independent calculations comprising the stated number of strands (x-axis) of which the average value and the standard deviation are shown as error bars. The thin dashed vertical line indicates the number of strands per pool used throughout this study. For (a), two identically calculated data sets (solid and dashed) with three repetitions each are shown to illustrate the fluctuations of $\chi_M(C)$ with less than 10^4 strands.

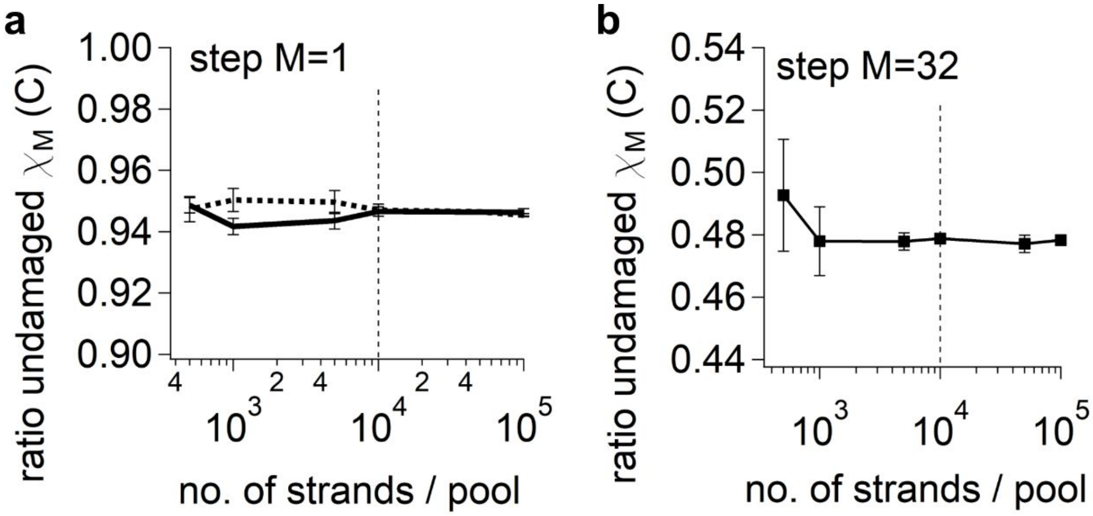

Fig. S2. Ratio of surviving strands for proto-genome pools plotted separately for all criteria used in Fig. 4C, specified in Table S2. For codon chronologies that contain ambiguously sortable subsets, the limit plots for the chronologies compatible with the corresponding minimum and maximum UV sensitivity chronologies are shown using the same color.

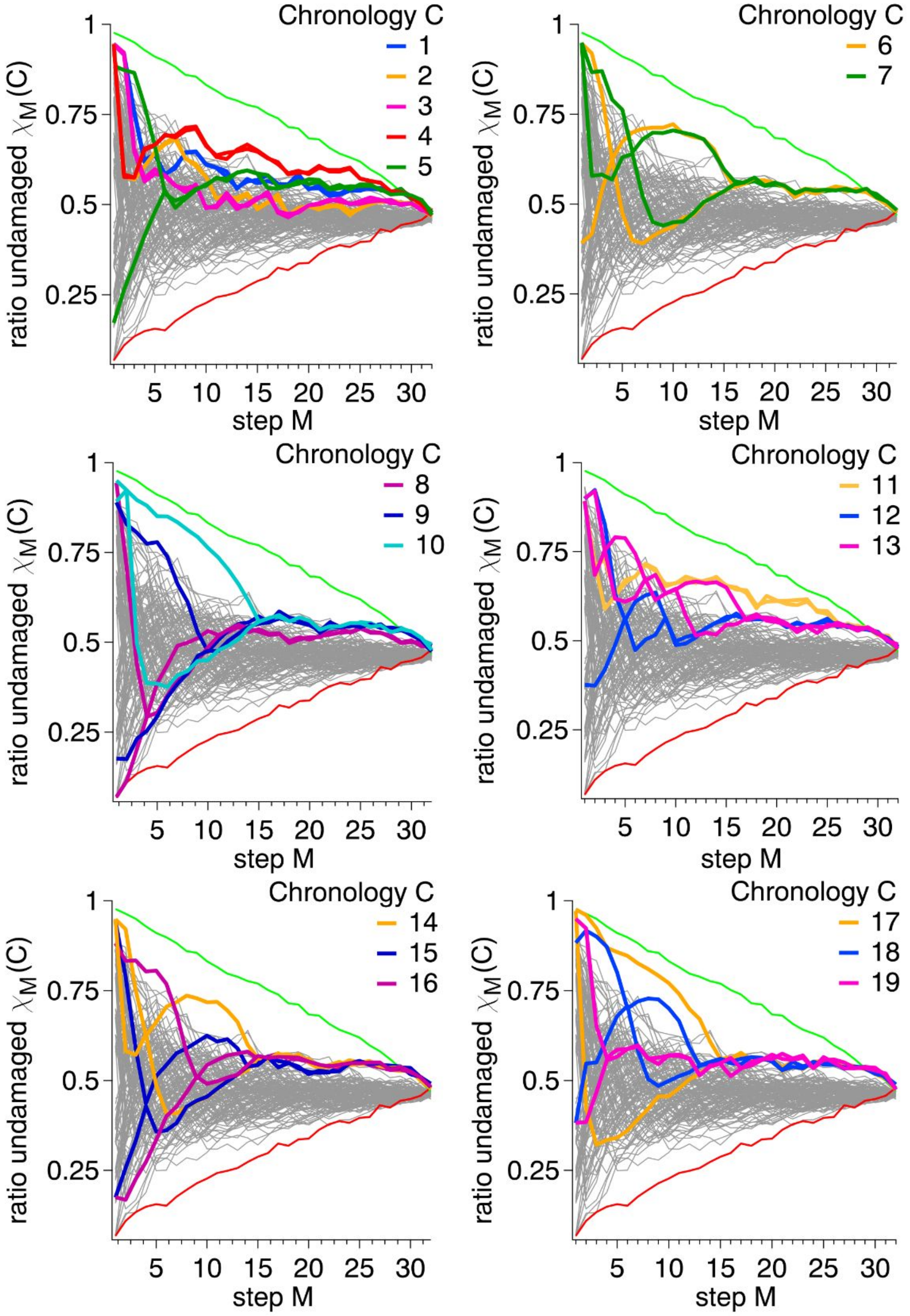

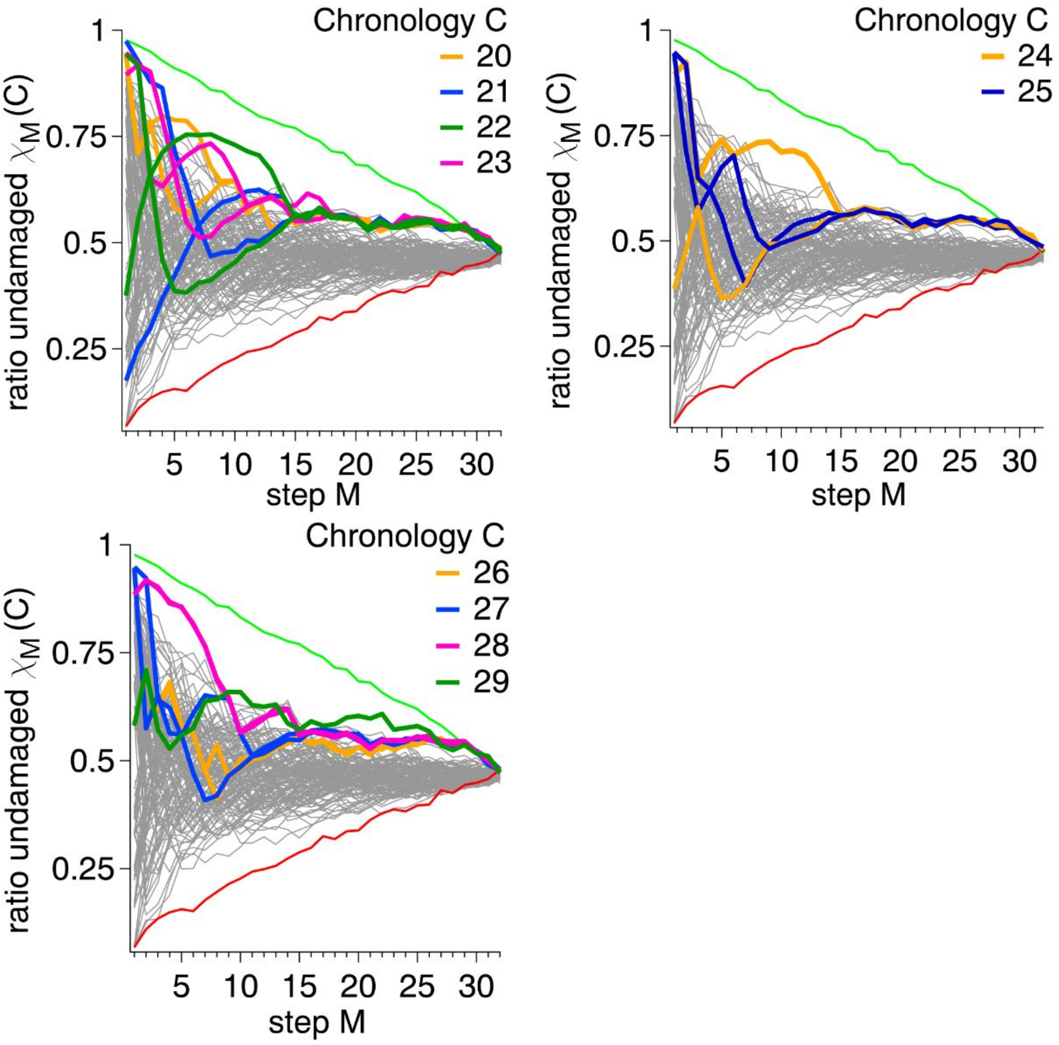

Chronology C
20
21
22
23
24
25
26
27
28
29
ratio undamaged χ_M(C)
1
0.75
0.5
0.25
5
10
15
20
25
30
step M

Figure S3. The average proportion of all 16 possible dimer sequences (y-axis) to occur at least once in a 150mer against the developmental step M for 100 randomly chosen codon chronologies. The proportion was calculated for 1000 strands and then normalized for the number of strands. After 4 to 5 steps, most of the dimer sequences occur in the 150mer protogenomes, so only small changes in UV stability χ are possible in the following steps, as shown in main text Figures 3A and 4A,B.

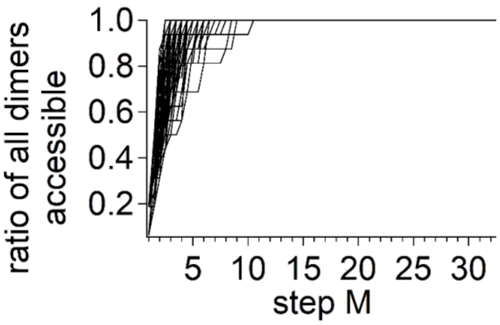

Figure S4. Comparison of the ratio of undamaged strands between sequence-averaged (a) and base-specific (b) photon absorption. For a detailed description, refer to SI appendix section 5. Chronologies (C) are defined in Table S2.

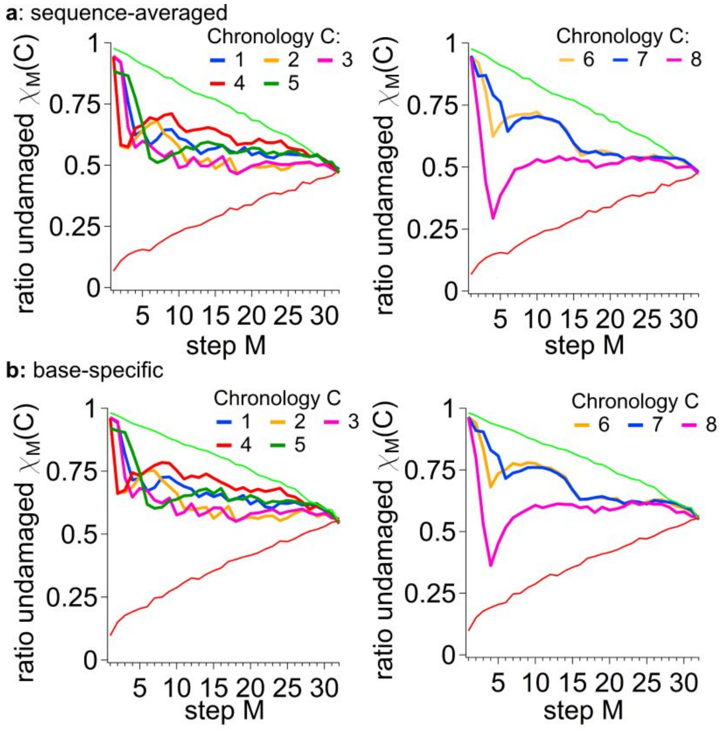

Table S1. Damage rates μ_i for all possible trimer sequences (see main text Fig. 1B,C).

3-mer	μ_i (10^{-3} dmg/photon)	3-mer	μ_i (10^{-3} dmg/photon)	3-mer	μ_i (10^{-3} dmg/photon)	3-mer	μ_i (10^{-3} dmg/photon)
AAA	7.2	TAA	12.0	GAA	4.4	CAA	5.1
AAC	4.8	TAC	13.1	GAC	2.8	CAC	8.3
AAG	3.7	TAG	6.5	GAG	1.8	CAG	2.5
AAT	11.8	TAT	18.1	GAT	7.5	CAT	14.3
ACA	3.9	TCA	18.2	GCA	3.1	CCA	14.1
ACC	11.6	TCC	26.1	GCC	11.0	CCC	22.5
ACG	2.8	TCG	14.2	GCG	1.6	CCG	11.1
ACT	18.3	TCT	34.5	GCT	15.4	CCT	28.3
AGA	3.2	TGA	7.7	GGA	2.9	CGA	4.3
AGC	3.1	TGC	9.6	GGC	2.5	CGC	9.4
AGG	2.4	TGG	5.4	GGG	1.1	CGG	3.0
AGT	3.8	TGT	9.0	GGT	4.2	CGT	7.7
ATA	3.8	TTA	28.6	GTA	2.6	CTA	19.1
ATC	13.2	TTC	39.4	GTC	10.4	CTC	30.4
ATG	2.4	TTG	19.6	GTG	0.1	CTG	8.9
ATT	29.6	TTT	57.6	GTT	16.7	CTT	39.1

Table S2. Amino acid chronologies and their calculated limit chronologies obtained by the algorithm described in SI appendix section 6. Chronologies are called unambiguous if a unique codon pair is selected in each step. For chronologies in which a specific amino acid can be selected interchangeably in one step from a group of amino acids indicated by brackets in the "AA-chronology" series, the chronologies are given with the minimum (min) and maximum (max) UV sensitivity according to the algorithm described in section 6 of the SI Appendix.

Chronology C	1	2	3	4	
Reference	Trifonov[14]	Haig[16], Wong[17], Papentin[18], Dufton[19]	Arques[20], Brooks[21,22], Eck[23]	Xia[24]	
AA chronology	GADVPSETLR NIQHKCFYMW	GASPVCDTNLKIE	AGVSDLTPEIR KNQHFMYCW	AGSPRDTCE (VW)HL(MQ)INYFK	
UV sensitivity	unambiguous	unambiguous	unambiguous	min	max
Codon chronology	GGC,GCC	GGC,GCC	GGC,GCC	GGC,GCC	GGC,GCC
	GAC,GTC	GGA,TCC	GAC,GTC	GGA,TCC	GGA,TCC
	GGG,CCC	GGG,CCC	GGA,TCC	GGG,CCC	GGG,CCC
	GGA,TCC	GCT,AGC	GAG,CTC	GCG,CGC	GCG,CGC
	GCT,AGC	GAC,GTC	GGT,ACC	GCT,AGC	GCT,AGC
	GAG,CTC	GCA,TGC	GGG,CCC	GAC,GTC	GAC,GTC
	GGT,ACC	GGT,ACC	TCA,TGA	GGT,ACC	GGT,ACC
	GCG,CGC	TCA,TGA	ACT,AGT	GCA,TGC	GCA,TGC
	CCG,CGG	GTT,AAC	ACA,TGT	CCG,CGG	CCG,CGG
	CCT,AGG	GAG,CTC	GAA,TTC	CCT,AGG	CCT,AGG
	TCG,CGA	CTT,AAG	GAT,ATC	GAG,CTC	GAG,CTC
	TCA,TGA	ACT,AGT	GCG,CGC	GTG,CAC	CCA,TGG
	TCT,AGA	GAT,ATC	CTT,AAG	CCA,TGG	GTG,CAC
	ACG,CGT	ACA,TGT	GCT,AGC	ATG,CAT	ATG,CAT
	ACT,AGT	GAA,TTC	GTG,CAC	TCG,CGA	TCG,CGA
	ACA,TGT	ATG,CAT	CCG,CGG	GTT,AAC	GTT,AAC
	GTT,AAC	GCG,CGC	TTT,AAA	TCA,TGA	TCA,TGA
	GAT,ATC	TTT,AAA	CCT,AGG	TCT,AGA	TCT,AGA
	GTG,CAC	CCG,CGG	ATG,CAT	ACG,CGT	ACG,CGT
	CTT,AAG	GTA,TAC	GTA,TAC	ACT,AGT	ACT,AGT
	GCA,TGC	CCT,AGG	GCA,TGC	ACA,TGT	ACA,TGT
	GAA,TTC	CCA,TGG	CCA,TGG	GAT,ATC	GAT,ATC
	CTG,CAG	TCG,CGA	TCG,CGA	CTG,CAG	CTG,CAG
	GTA,TAC	TCT,AGA	TCT,AGA	GTA,TAC	GTA,TAC
	ATG,CAT	ACG,CGT	ACG,CGT	GAA,TTC	GAA,TTC
	CCA,TGG	GTG,CAC	CTG,CAG	CTA,TAG	CTA,TAG
	CTA,TAG	CTG,CAG	CTA,TAG	CAA,TTG	CAA,TTG
	CAA,TTG	CTA,TAG	GTT,AAC	CTT,AAG	CTT,AAG
	ATA,TAT	CAA,TTG	CAA,TTG	ATA,TAT	ATA,TAT
	ATT,AAT	ATA,TAT	ATA,TAT	ATT,AAT	ATT,AAT
	TTA,TAA	ATT,AAT	ATT,AAT	TTA,TAA	TTA,TAA
	TTT,AAA	TTA,TAA	TTA,TAA	TTT,AAA	TTT,AAA

5		6		7		8	
Osawa[25]		Crick[26]		Eigen[27,28]		Ferreira[29]	
(ADEFGHP)VSTLRI Q(KNY)(MW)C		(DGNS)(ACEFHIK LMPQRTVWY)		(AG)(DINSTV)(CE FHKLMPQRWY)		(FGKLNP)(CDEH QRSTVW)(AIMY)	
min	max	min	max	min	max	min	max
GTG,CAC	GAA,TTC	GGC,GCC	GGA,TCC	GGC,GCC	GGC,GCC	GGC,GCC	TTT,AAA
GGC,GCC	GAG,CTC	GAC,GTC	GTT,AAC	GGT,ACC	GGA,TCC	GGG,CCC	GAA,TTC
GAC,GTC	GGG,CCC	GTT,AAC	GAC,GTC	GAC,GTC	GAT,ATC	GAA,TTC	GGG,CCC
GGG,CCC	GAC,GTC	GGA,TCC	GGC,GCC	GTT,AAC	GTT,AAC	TTT,AAA	GGC,GCC
GAG,CTC	GGC,GCC	GCG,CGC	GAA,TTC	GAT,ATC	GAC,GTC	GCG,CGC	GGA,TCC
GAA,TTC	GTG,CAC	GTG,CAC	CTT,AAG	GGA,TCC	GGT,ACC	GTG,CAC	GAC,GTC
GGA,TCC	GGA,TCC	GCA,TGC	GAG,CTC	GCG,CGC	GAA,TTC	GCA,TGC	CCA,TGG
GCT,AGC	GCT,AGC	ATG,CAT	GAT,ATC	GTG,CAC	CTT,AAG	CCA,TGG	GGT,ACC
GGT,ACC	GGT,ACC	GTA,TAC	CCA,TGG	ATG,CAT	GAG,CTC	GGT,ACC	GCA,TGC
CTG,CAG	CTG,CAG	GGT,ACC	GGG,CCC	GCA,TGC	GGG,CCC	GAC,GTC	GTG,CAC
GCG,CGC	GCG,CGC	CCA,TGG	GGT,ACC	GTA,TAC	CCA,TGG	GGA,TCC	GCG,CGC
GAT,ATC	GAT,ATC	GGG,CCC	GTA,TAC	CCA,TGG	GTA,TAC	GCT,AGC	GCT,AGC
CCG,CGG	CCG,CGG	GAT,ATC	GCA,TGC	GGG,CCC	GTG,CAC	CCG,CGG	CCG,CGG
ATG,CAT	ATG,CAT	GAG,CTC	GTG,CAC	GAG,CTC	GCA,TGC	CCT,AGG	CCT,AGG
CCT,AGG	CCT,AGG	GAA,TTC	ATG,CAT	CTT,AAG	GCG,CGC	TCG,CGA	TCG,CGA
GTA,TAC	CTT,AAG	CTT,AAG	GCG,CGC	GAA,TTC	ATG,CAT	GAG,CTC	GAG,CTC
CTT,AAG	GTA,TAC	GCT,AGC	GCT,AGC	GCT,AGC	GCT,AGC	TCA,TGA	TCA,TGA
CCA,TGG	CCA,TGG	CCG,CGG	CCG,CGG	CCG,CGG	CCG,CGG	TCT,AGA	TCT,AGA
GCA,TGC	GCA,TGC	CCT,AGG	CCT,AGG	CCT,AGG	CCT,AGG	ACG,CGT	ACG,CGT
TCG,CGA	TCG,CGA	TCG,CGA	TCG,CGA	TCG,CGA	TCG,CGA	ACT,AGT	ACT,AGT
TCA,TGA	TCA,TGA	TCA,TGA	TCA,TGA	TCA,TGA	TCA,TGA	ACA,TGT	ACA,TGT
TCT,AGA	TCT,AGA	TCT,AGA	TCT,AGA	TCT,AGA	TCT,AGA	ATG,CAT	GAT,ATC
ACG,CGT	ACG,CGT	ACG,CGT	ACG,CGT	ACG,CGT	ACG,CGT	GTA,TAC	GTA,TAC
ACT,AGT	ACT,AGT	ACT,AGT	ACT,AGT	ACT,AGT	ACT,AGT	GAT,ATC	ATG,CAT
ACA,TGT	ACA,TGT	ACA,TGT	ACA,TGT	ACA,TGT	ACA,TGT	CTG,CAG	CTG,CAG
CTA,TAG	CTA,TAG	CTG,CAG	CTG,CAG	CTG,CAG	CTG,CAG	CTA,TAG	CTA,TAG
GTT,AAC	GTT,AAC	CTA,TAG	CTA,TAG	CTA,TAG	CTA,TAG	GTT,AAC	GTT,AAC
CAA,TTG	CAA,TTG	CAA,TTG	CAA,TTG	CAA,TTG	CAA,TTG	CAA,TTG	CAA,TTG
ATA,TAT	ATA,TAT	ATA,TAT	ATA,TAT	ATA,TAT	ATA,TAT	CTT,AAG	CTT,AAG
ATT,AAT	ATT,AAT	ATT,AAT	ATT,AAT	ATT,AAT	ATT,AAT	ATA,TAT	ATA,TAT
TTA,TAA	TTA,TAA	TTA,TAA	TTA,TAA	TTA,TAA	TTA,TAA	ATT,AAT	ATT,AAT
TTT,AAA	TTT,AAA	TTT,AAA	TTT,AAA	TTT,AAA	TTT,AAA	TTA,TAA	TTA,TAA

9		10		11		12	
Nelsestuen[30]		Möller[31]		Chaley[32]		Henikoff[33]	
(DEFHIKLMSTVY) (ACGNPQRW)		(ADGV)(CEFHIK LMNPQRSTWY)		QHP(LS)GCWR V(DE)AYT(IM)F(KN)		(AILSV)(EKMQRT) (DFGN)(PY)HCW	
min	max	min	max	min	max	min	max
GTG,CAC	CTT,AAG	GAC,GTC	GGC,GCC	GTG,CAC	GTG,CAC	GAC,GTC	GAG,CTC
ATG,CAT	GAA,TTC	GGC,GCC	GAC,GTC	GGG,CCC	GGG,CCC	GGC,GCC	GGA,TCC
GTA,TAC	GAG,CTC	GCG,CGC	GAA,TTC	CTG,CAG	GGA,TCC	GAT,ATC	GAT,ATC
GGT,ACC	GGA,TCC	GTG,CAC	CTT,AAG	GGA,TCC	CTG,CAG	GGA,TCC	GAC,GTC
GAC,GTC	GAT,ATC	ATG,CAT	GAG,CTC	GCA,TGC	GCA,TGC	GAG,CTC	GGC,GCC
GAT,ATC	GAC,GTC	GCA,TGC	GGA,TCC	CCA,TGG	CCA,TGG	GCG,CGC	CTT,AAG
GGA,TCC	GGT,ACC	GTA,TAC	GAT,ATC	GCG,CGC	GCG,CGC	ATG,CAT	GGT,ACC
GAG,CTC	GTA,TAC	GGT,ACC	GGG,CCC	GAC,GTC	GAC,GTC	GGT,ACC	ATG,CAT
GAA,TTC	GTG,CAC	CCA,TGG	GGT,ACC	GAG,CTC	GAG,CTC	CTT,AAG	GCG,CGC
CTT,AAG	ATG,CAT	GGG,CCC	CCA,TGG	GGC,GCC	GGC,GCC	GAA,TTC	GAA,TTC
GCG,CGC	GGG,CCC	GAT,ATC	GTA,TAC	GTA,TAC	GTA,TAC	GTA,TAC	GGG,CCC
GCA,TGC	CCA,TGG	GGA,TCC	ATG,CAT	GCT,AGC	GCT,AGC	GGG,CCC	GTA,TAC
GGC,GCC	GGC,GCC	GAG,CTC	GCA,TGC	GGT,ACC	GGT,ACC	GTG,CAC	GTG,CAC
CCA,TGG	GCG,CGC	GAA,TTC	GCG,CGC	CCG,CGG	CCG,CGG	GCT,AGC	GCT,AGC
GGG,CCC	GCA,TGC	CTT,AAG	GTG,CAC	CCT,AGG	CCT,AGG	GCA,TGC	GCA,TGC
GCT,AGC	GCT,AGC	GCT,AGC	GCT,AGC	TCG,CGA	TCG,CGA	CCG,CGG	CCG,CGG
CCG,CGG	CCG,CGG	CCG,CGG	CCG,CGG	TCA,TGA	TCA,TGA	CCT,AGG	CCT,AGG
CCT,AGG	CCT,AGG	CCT,AGG	CCT,AGG	TCT,AGA	TCT,AGA	CCA,TGG	CCA,TGG
TCG,CGA	TCG,CGA	TCG,CGA	TCG,CGA	ACG,CGT	ACG,CGT	TCG,CGA	TCG,CGA
TCA,TGA	TCA,TGA	TCA,TGA	TCA,TGA	ACT,AGT	ACT,AGT	TCA,TGA	TCA,TGA
TCT,AGA	TCT,AGA	TCT,AGA	TCT,AGA	ACA,TGT	ACA,TGT	TCT,AGA	TCT,AGA
ACG,CGT	ACG,CGT	ACG,CGT	ACG,CGT	ATG,CAT	GAT,ATC	ACG,CGT	ACG,CGT
ACT,AGT	ACT,AGT	ACT,AGT	ACT,AGT	GAT,ATC	ATG,CAT	ACT,AGT	ACT,AGT
ACA,TGT	ACA,TGT	ACA,TGT	ACA,TGT	GAA,TTC	GAA,TTC	ACA,TGT	ACA,TGT
CTG,CAG	CTG,CAG	CTG,CAG	CTG,CAG	CTA,TAG	CTA,TAG	CTG,CAG	CTG,CAG
CTA,TAG	CTA,TAG	CTA,TAG	CTA,TAG	CTT,AAG	CTT,AAG	CTA,TAG	CTA,TAG
GTT,AAC	GTT,AAC	GTT,AAC	GTT,AAC	GTT,AAC	GTT,AAC	GTT,AAC	GTT,AAC
CAA,TTG	CAA,TTG	CAA,TTG	CAA,TTG	CAA,TTG	CAA,TTG	CAA,TTG	CAA,TTG
ATA,TAT	ATA,TAT	ATA,TAT	ATA,TAT	ATA,TAT	ATA,TAT	ATA,TAT	ATA,TAT
ATT,AAT	ATT,AAT	ATT,AAT	ATT,AAT	ATT,AAT	ATT,AAT	ATT,AAT	ATT,AAT
TTA,TAA	TTA,TAA	TTA,TAA	TTA,TAA	TTA,TAA	TTA,TAA	TTA,TAA	TTA,TAA
TTT,AAA	TTT,AAA	TTT,AAA	TTT,AAA	TTT,AAA	TTT,AAA	TTT,AAA	TTT,AAA

13		14		15		16	
Eigen[34]		Kvenvolden[35]		Riddle[36]		Arques[20]	
V(AGP)(ENRT)(LQS) (CDFHIKMYW)		(AG)(DEPV)(CFHIK LMNQRSTWY)		(AGEIK)(CDFHLM NPQRSTVWY)		(ADEFGIKLNQTVY) (CHMPRSW)	
min	max	min	max	min	max	min	max
GAC,GTC	GAC,GTC	GGC,GCC	GGC,GCC	GGC,GCC	CTT,AAG	GTG,CAC	CTT,AAG
GGC,GCC	GGG,CCC	GAC,GTC	GAG,CTC	GAT,ATC	GAG,CTC	GTA,TAC	GAA,TTC
GGG,CCC	GGC,GCC	GGG,CCC	GGG,CCC	GAG,CTC	GAT,ATC	GGC,GCC	GAG,CTC
GCG,CGC	GAG,CTC	GAG,CTC	GAC,GTC	CTT,AAG	GGC,GCC	GGT,ACC	GAT,ATC
GGT,ACC	GTT,AAC	GTG,CAC	GAA,TTC	GCG,CGC	GAA,TTC	GAC,GTC	GGT,ACC
GTT,AAC	GGT,ACC	GCG,CGC	CTT,AAG	ATG,CAT	GGA,TCC	GAT,ATC	GAC,GTC
GAG,CTC	GCG,CGC	ATG,CAT	GGA,TCC	GCA,TGC	GGG,CCC	GAG,CTC	GTA,TAC
GTG,CAC	GGA,TCC	GCA,TGC	GAT,ATC	GTA,TAC	GAC,GTC	CTT,AAG	GGC,GCC
GGA,TCC	GTG,CAC	GTA,TAC	CCA,TGG	GGT,ACC	GGT,ACC	GAA,TTC	GTG,CAC
GCT,AGC	GCT,AGC	CCA,TGG	GGT,ACC	GAC,GTC	CCA,TGG	GCG,CGC	GGA,TCC
GCA,TGC	CTT,AAG	GGT,ACC	GTA,TAC	CCA,TGG	GTA,TAC	GCA,TGC	GGG,CCC
ATG,CAT	GAA,TTC	GAT,ATC	GCA,TGC	GGG,CCC	GCG,CGC	ATG,CAT	CCA,TGG
GTA,TAC	GAT,ATC	GGA,TCC	ATG,CAT	GGA,TCC	ATG,CAT	CCA,TGG	GCA,TGC
CCA,TGG	CCA,TGG	GAA,TTC	GTG,CAC	GAA,TTC	GCA,TGC	GGG,CCC	ATG,CAT
GAT,ATC	GCA,TGC	CTT,AAG	GCG,CGC	GCT,AGC	GCT,AGC	GGA,TCC	GCG,CGC
GAA,TTC	GTA,TAC	GCT,AGC	GCT,AGC	CCG,CGG	CCG,CGG	GCT,AGC	GCT,AGC
CTT,AAG	ATG,CAT	CCG,CGG	CCG,CGG	CCT,AGG	CCT,AGG	CCG,CGG	CCG,CGG
CCG,CGG	CCG,CGG	CCT,AGG	CCT,AGG	TCG,CGA	TCG,CGA	CCT,AGG	CCT,AGG
CCT,AGG	CCT,AGG	TCG,CGA	TCG,CGA	TCA,TGA	TCA,TGA	TCG,CGA	TCG,CGA
TCG,CGA	TCG,CGA	TCA,TGA	TCA,TGA	TCT,AGA	TCT,AGA	TCA,TGA	TCA,TGA
TCA,TGA	TCA,TGA	TCT,AGA	TCT,AGA	ACG,CGT	ACG,CGT	TCT,AGA	TCT,AGA
TCT,AGA	TCT,AGA	ACG,CGT	ACG,CGT	ACT,AGT	ACT,AGT	ACG,CGT	ACG,CGT
ACG,CGT	ACG,CGT	ACT,AGT	ACT,AGT	ACA,TGT	ACA,TGT	ACT,AGT	ACT,AGT
ACT,AGT	ACT,AGT	ACA,TGT	ACA,TGT	GTG,CAC	GTG,CAC	ACA,TGT	ACA,TGT
ACA,TGT	ACA,TGT	CTG,CAG	CTG,CAG	CTG,CAG	CTG,CAG	CTG,CAG	CTG,CAG
CTG,CAG	CTG,CAG	CTA,TAG	CTA,TAG	CTA,TAG	CTA,TAG	CTA,TAG	CTA,TAG
CTA,TAG	CTA,TAG	GTT,AAC	GTT,AAC	GTT,AAC	GTT,AAC	GTT,AAC	GTT,AAC
CAA,TTG	CAA,TTG	CAA,TTG	CAA,TTG	CAA,TTG	CAA,TTG	CAA,TTG	CAA,TTG
ATA,TAT	ATA,TAT	ATA,TAT	ATA,TAT	ATA,TAT	ATA,TAT	ATA,TAT	ATA,TAT
ATT,AAT	ATT,AAT	ATT,AAT	ATT,AAT	ATT,AAT	ATT,AAT	ATT,AAT	ATT,AAT
TTA,TAA	TTA,TAA	TTA,TAA	TTA,TAA	TTA,TAA	TTA,TAA	TTA,TAA	TTA,TAA
TTT,AAA	TTT,AAA	TTT,AAA	TTT,AAA	TTT,AAA	TTT,AAA	TTT,AAA	TTT,AAA

17		18		19		20	
Yarus[37]		Jukes[25]		Wong[17]		Hartman[38]	
R(ACDEFGHIKL MNPQSTVWY)		(ADEGHLPQRV) (CFIKNSTY)(MW)		(ADEGS)V(PT)(IL) FCY(KR)(NQ)H(MW)		GPAR(DENQST)(H K)C(FILVY)(MW)	
min	max	min	max	min	max	min	max
GCG,CGC	GCG,CGC	GTG,CAC	GAG,CTC	GGC,GCC	GGA,TCC	GGC,GCC	GGC,GCC
GCA,TGC	CTT,AAG	GCG,CGC	GAC,GTC	GAC,GTC	GAG,CTC	GGG,CCC	GGG,CCC
GTG,CAC	GAA,TTC	GGC,GCC	GGG,CCC	GGA,TCC	GAC,GTC	GCG,CGC	GCG,CGC
ATG,CAT	GAG,CTC	GAC,GTC	GGC,GCC	GAG,CTC	GGC,GCC	GTG,CAC	GAG,CTC
GTA,TAC	GGA,TCC	GGG,CCC	GCG,CGC	GGT,ACC	GGG,CCC	GGT,ACC	GGA,TCC
GGC,GCC	GAT,ATC	GAG,CTC	GTG,CAC	GGG,CCC	GGT,ACC	GAC,GTC	GTT,AAC
CCA,TGG	GGG,CCC	GCA,TGC	GAA,TTC	GCT,AGC	GCT,AGC	GTT,AAC	GAC,GTC
GGT,ACC	GAC,GTC	GTA,TAC	CTT,AAG	TCA,TGA	TCA,TGA	GGA,TCC	GGT,ACC
GAC,GTC	GGT,ACC	GGT,ACC	GGA,TCC	ACT,AGT	ACT,AGT	GAG,CTC	GTG,CAC
GGG,CCC	CCA,TGG	GAT,ATC	GAT,ATC	ACA,TGT	ACA,TGT	GCT,AGC	GCT,AGC
GAT,ATC	GTA,TAC	GGA,TCC	GTA,TAC	CTG,CAG	GAT,ATC	CTT,AAG	CTT,AAG
GGA,TCC	GCA,TGC	CTT,AAG	GGT,ACC	GAT,ATC	CTG,CAG	GCA,TGC	GCA,TGC
GAG,CTC	GGC,GCC	GAA,TTC	GCA,TGC	GAA,TTC	GAA,TTC	GTA,TAC	GAA,TTC
GAA,TTC	ATG,CAT	GCT,AGC	GCT,AGC	GCA,TGC	GCA,TGC	GAT,ATC	GAT,ATC
CTT,AAG	GTG,CAC	CCG,CGG	CCG,CGG	GTA,TAC	GTA,TAC	GAA,TTC	GTA,TAC
GCT,AGC	GCT,AGC	CCT,AGG	CCT,AGG	GCG,CGC	CTT,AAG	CCG,CGG	CCG,CGG
CCG,CGG	CCG,CGG	ATG,CAT	CCA,TGG	CTT,AAG	GCG,CGC	ATG,CAT	CCA,TGG
CCT,AGG	CCT,AGG	CCA,TGG	ATG,CAT	GTG,CAC	GTG,CAC	CCA,TGG	ATG,CAT
TCG,CGA	TCG,CGA	TCG,CGA	TCG,CGA	CCG,CGG	CCG,CGG	CCT,AGG	CCT,AGG
TCA,TGA	TCA,TGA	TCA,TGA	TCA,TGA	ATG,CAT	CCA,TGG	TCG,CGA	TCG,CGA
TCT,AGA	TCT,AGA	TCT,AGA	TCT,AGA	CCA,TGG	ATG,CAT	TCA,TGA	TCA,TGA
ACG,CGT	ACG,CGT	ACG,CGT	ACG,CGT	CCT,AGG	CCT,AGG	TCT,AGA	TCT,AGA
ACT,AGT	ACT,AGT	ACT,AGT	ACT,AGT	TCG,CGA	TCG,CGA	ACG,CGT	ACG,CGT
ACA,TGT	ACA,TGT	ACA,TGT	ACA,TGT	TCT,AGA	TCT,AGA	ACT,AGT	ACT,AGT
CTG,CAG	CTG,CAG	CTG,CAG	CTG,CAG	ACG,CGT	ACG,CGT	ACA,TGT	ACA,TGT
CTA,TAG	CTA,TAG	CTA,TAG	CTA,TAG	CTA,TAG	CTA,TAG	CTG,CAG	CTG,CAG
GTT,AAC	GTT,AAC	GTT,AAC	GTT,AAC	GTT,AAC	GTT,AAC	CTA,TAG	CTA,TAG
CAA,TTG	CAA,TTG	CAA,TTG	CAA,TTG	CAA,TTG	CAA,TTG	CAA,TTG	CAA,TTG
ATA,TAT	ATA,TAT	ATA,TAT	ATA,TAT	ATA,TAT	ATA,TAT	ATA,TAT	ATA,TAT
ATT,AAT	ATT,AAT	ATT,AAT	ATT,AAT	ATT,AAT	ATT,AAT	ATT,AAT	ATT,AAT
TTA,TAA	TTA,TAA	TTA,TAA	TTA,TAA	TTA,TAA	TTA,TAA	TTA,TAA	TTA,TAA
TTT,AAA	TTT,AAA	TTT,AAA	TTT,AAA	TTT,AAA	TTT,AAA	TTT,AAA	TTT,AAA

21		22		23		24	
N16 in Trifonov[14]		Yarus[37]		Taylor[39]		Ikehara[40]	
(ADEGKRSTV)(CFHILMNPQWY)		(ADEGV)(CFHIKLMNPQRSTWY)		(ADGV)(LPR)(CIKQST)(EFHMNWY)		(ADGV)E(HLPQR)(CFIKMNSTWY)	
min	max	min	max	min	max	min	max
GCG,CGC	CTT,AAG	GGC,GCC	GAG,CTC	GAC,GTC	GGC,GCC	GAC,GTC	GGC,GCC
GGC,GCC	GAG,CTC	GAC,GTC	GAC,GTC	GGC,GCC	GAC,GTC	GGC,GCC	GAC,GTC
GGT,ACC	GGA,TCC	GAG,CTC	GGC,GCC	GCG,CGC	GAG,CTC	GAG,CTC	GAG,CTC
GAC,GTC	GGT,ACC	GCG,CGC	GAA,TTC	GGG,CCC	GGG,CCC	GCG,CGC	GGG,CCC
GGA,TCC	GAC,GTC	GTG,CAC	CTT,AAG	GAG,CTC	GCG,CGC	GTG,CAC	GCG,CGC
GAG,CTC	GGC,GCC	GCA,TGC	GGA,TCC	GTG,CAC	CTT,AAG	GGG,CCC	GTG,CAC
CTT,AAG	GCG,CGC	ATG,CAT	GAT,ATC	GCA,TGC	GGA,TCC	GCA,TGC	CTT,AAG
GTG,CAC	GAA,TTC	GTA,TAC	GGG,CCC	GGT,ACC	GAT,ATC	ATG,CAT	GAA,TTC
ATG,CAT	GAT,ATC	CCA,TGG	CCA,TGG	GAT,ATC	GGT,ACC	GTA,TAC	GGA,TCC
GCA,TGC	GGG,CCC	GGT,ACC	GGT,ACC	GGA,TCC	GCA,TGC	CCA,TGG	GAT,ATC
GTA,TAC	GTA,TAC	GGG,CCC	GTA,TAC	CTT,AAG	GTG,CAC	GGT,ACC	CCA,TGG
CCA,TGG	CCA,TGG	GAT,ATC	ATG,CAT	GCT,AGC	GCT,AGC	GAT,ATC	GGT,ACC
GGG,CCC	GCA,TGC	GGA,TCC	GCA,TGC	CCG,CGG	CCG,CGG	GGA,TCC	GTA,TAC
GAT,ATC	GTG,CAC	GAA,TTC	GTG,CAC	CCT,AGG	CCT,AGG	GAA,TTC	GCA,TGC
GAA,TTC	ATG,CAT	CTT,AAG	GCG,CGC	ATG,CAT	GAA,TTC	CTT,AAG	ATG,CAT
GCT,AGC	GCT,AGC	GCT,AGC	GCT,AGC	GTA,TAC	CCA,TGG	GCT,AGC	GCT,AGC
CCG,CGG	CCG,CGG	CCG,CGG	CCG,CGG	CCA,TGG	GTA,TAC	CCG,CGG	CCG,CGG
CCT,AGG	CCT,AGG	CCT,AGG	CCT,AGG	GAA,TTC	ATG,CAT	CCT,AGG	CCT,AGG
TCG,CGA	TCG,CGA	TCG,CGA	TCG,CGA	TCG,CGA	TCG,CGA	TCG,CGA	TCG,CGA
TCA,TGA	TCA,TGA	TCA,TGA	TCA,TGA	TCA,TGA	TCA,TGA	TCA,TGA	TCA,TGA
TCT,AGA	TCT,AGA	TCT,AGA	TCT,AGA	TCT,AGA	TCT,AGA	TCT,AGA	TCT,AGA
ACG,CGT	ACG,CGT	ACG,CGT	ACG,CGT	ACG,CGT	ACG,CGT	ACG,CGT	ACG,CGT
ACT,AGT	ACT,AGT	ACT,AGT	ACT,AGT	ACT,AGT	ACT,AGT	ACT,AGT	ACT,AGT
ACA,TGT	ACA,TGT	ACA,TGT	ACA,TGT	ACA,TGT	ACA,TGT	ACA,TGT	ACA,TGT
CTG,CAG	CTG,CAG	CTG,CAG	CTG,CAG	CTG,CAG	CTG,CAG	CTG,CAG	CTG,CAG
CTA,TAG	CTA,TAG	CTA,TAG	CTA,TAG	CTA,TAG	CTA,TAG	CTA,TAG	CTA,TAG
GTT,AAC	GTT,AAC	GTT,AAC	GTT,AAC	GTT,AAC	GTT,AAC	GTT,AAC	GTT,AAC
CAA,TTG	CAA,TTG	CAA,TTG	CAA,TTG	CAA,TTG	CAA,TTG	CAA,TTG	CAA,TTG
ATA,TAT	ATA,TAT	ATA,TAT	ATA,TAT	ATA,TAT	ATA,TAT	ATA,TAT	ATA,TAT
ATT,AAT	ATT,AAT	ATT,AAT	ATT,AAT	ATT,AAT	ATT,AAT	ATT,AAT	ATT,AAT
TTA,TAA	TTA,TAA	TTA,TAA	TTA,TAA	TTA,TAA	TTA,TAA	TTA,TAA	TTA,TAA
TTT,AAA	TTT,AAA	TTT,AAA	TTT,AAA	TTT,AAA	TTT,AAA	TTT,AAA	TTT,AAA

25		26		27		28	
N54 in Trifonov[14]		N55 in Trifonov[14]		N57 in Trifonov[14]		Baumann[41]	
(GPS)(DEFKLN) (AHQRV)(CIMTWY)		GSDNK(AF)(HT)E QL(PV)R(CW)(IM)Y		G(DS)A(LPV)(EFH IKMNQR)(CTWY)		(ADEGILPQRSTV) (KN)(CFHY)(MW)	
min	max	min	max	min	max	min	max
GGC,GCC	GGA,TCC	GGC,GCC	GGC,GCC	GGC,GCC	GGC,GCC	GTG,CAC	GAG,CTC
GGG,CCC	GGG,CCC	GGA,TCC	GGA,TCC	GAC,GTC	GGA,TCC	GCG,CGC	GGA,TCC
GGA,TCC	GGC,GCC	GCT,AGC	GCT,AGC	GGA,TCC	GAC,GTC	GGC,GCC	GAT,ATC
GAC,GTC	CTT,AAG	GAC,GTC	GAC,GTC	GGG,CCC	GAG,CTC	GGT,ACC	GGG,CCC
GAG,CTC	GAA,TTC	TCA,TGA	TCA,TGA	GAG,CTC	GGG,CCC	GAC,GTC	GGT,ACC
CTT,AAG	GAG,CTC	GTT,AAC	GTT,AAC	GTG,CAC	GAA,TTC	GAT,ATC	GAC,GTC
GAA,TTC	GAC,GTC	CTT,AAG	CTT,AAG	GCG,CGC	CTT,AAG	GGG,CCC	GGC,GCC
GTG,CAC	GCG,CGC	GCG,CGC	GAA,TTC	ATG,CAT	GAT,ATC	GGA,TCC	GCG,CGC
GCG,CGC	GTG,CAC	GAA,TTC	GCG,CGC	GAT,ATC	GTG,CAC	GAG,CTC	GTG,CAC
ATG,CAT	GAT,ATC	GTG,CAC	GGT,ACC	GAA,TTC	ATG,CAT	CTT,AAG	CTT,AAG
GCA,TGC	CCA,TGG	GGT,ACC	GTG,CAC	CTT,AAG	GCG,CGC	GCT,AGC	GCT,AGC
GTA,TAC	GGT,ACC	GGG,CCC	GGG,CCC	GCT,AGC	GCT,AGC	GCA,TGC	GAA,TTC
CCA,TGG	GTA,TAC	CCG,CGG	CCG,CGG	GCA,TGC	CCA,TGG	ATG,CAT	GTA,TAC
GGT,ACC	GCA,TGC	GCA,TGC	CCA,TGG	GTA,TAC	GTA,TAC	GTA,TAC	GCA,TGC
GAT,ATC	ATG,CAT	CCA,TGG	GCA,TGC	GGT,ACC	GGT,ACC	GAA,TTC	ATG,CAT
GCT,AGC	GCT,AGC	CCT,AGG	CCT,AGG	CCA,TGG	GCA,TGC	CCG,CGG	CCG,CGG
CCG,CGG	CCG,CGG	TCG,CGA	TCG,CGA	CCG,CGG	CCG,CGG	CCT,AGG	CCT,AGG
CCT,AGG	CCT,AGG	GAG,CTC	GAG,CTC	CCT,AGG	CCT,AGG	CCA,TGG	CCA,TGG
TCG,CGA	TCG,CGA	TCT,AGA	TCT,AGA	TCG,CGA	TCG,CGA	TCG,CGA	TCG,CGA
TCA,TGA	TCA,TGA	ACG,CGT	ACG,CGT	TCA,TGA	TCA,TGA	TCA,TGA	TCA,TGA
TCT,AGA	TCT,AGA	ACT,AGT	ACT,AGT	TCT,AGA	TCT,AGA	TCT,AGA	TCT,AGA
ACG,CGT	ACG,CGT	ACA,TGT	ACA,TGT	ACG,CGT	ACG,CGT	ACG,CGT	ACG,CGT
ACT,AGT	ACT,AGT	ATG,CAT	GAT,ATC	ACT,AGT	ACT,AGT	ACT,AGT	ACT,AGT
ACA,TGT	ACA,TGT	GAT,ATC	ATG,CAT	ACA,TGT	ACA,TGT	ACA,TGT	ACA,TGT
CTG,CAG	CTG,CAG	CTG,CAG	CTG,CAG	CTG,CAG	CTG,CAG	CTG,CAG	CTG,CAG
CTA,TAG	CTA,TAG	GTA,TAC	GTA,TAC	CTA,TAG	CTA,TAG	CTA,TAG	CTA,TAG
GTT,AAC	GTT,AAC	CTA,TAG	CTA,TAG	GTT,AAC	GTT,AAC	GTT,AAC	GTT,AAC
CAA,TTG	CAA,TTG	CAA,TTG	CAA,TTG	CAA,TTG	CAA,TTG	CAA,TTG	CAA,TTG
ATA,TAT	ATA,TAT	ATA,TAT	ATA,TAT	ATA,TAT	ATA,TAT	ATA,TAT	ATA,TAT
ATT,AAT	ATT,AAT	ATT,AAT	ATT,AAT	ATT,AAT	ATT,AAT	ATT,AAT	ATT,AAT
TTA,TAA	TTA,TAA	TTA,TAA	TTA,TAA	TTA,TAA	TTA,TAA	TTA,TAA	TTA,TAA
TTT,AAA	TTT,AAA	TTT,AAA	TTT,AAA	TTT,AAA	TTT,AAA	TTT,AAA	TTT,AAA

29
Li[42]
Direct codon chronology
Unambiguous
GGG,CCC<br>GGC,GCC<br>GGA,TCC<br>GAG,CTC<br>GAC,GTC<br>GGT,ACC<br>GCG,CGC<br>AGC,GCT<br>GCA,TGC<br>CGG,CCG<br>AGG,CCT<br>TGG,CCA<br>CGA,TCG<br>AGA,TCT<br>TGA,TCA<br>ACG,CGT<br>AGT,ACT<br>ACA,TGT<br>GTG,CAC<br>CAG,CTG<br>GAT,ATC<br>ATG,CAT<br>GAA,TTC<br>GTA,TAC<br>TAG,CTA<br>AAC,GTT<br>AAG,CTT<br>CAA,TTG<br>ATA,TAT<br>TAA,ATT<br>TAA,TTA<br>AAA,TTT

Table S3. Calculated number of effective doses

Sample #	1	2	3	4	5	6	7	8	9	10	11	12
Dose (photons/base)	2	15	22	31	45	63	92	127	184	261	370	559

Table S4. Molecular damage rates $\Phi_{i,j}$ for dimers: The rates for the formation of dimeric ions in AT, AC, GC, AG, GG and GT were below the detection limit and are reported as N.A..

Sequence i	TT	TC	CC	AA	AT	AC	GC	AG	GG	GT
Rate Φ_i (10^{-3} dmg/photon)	20	10	5	2	N.A.	N.A.	N.A.	N.A.	N.A.	N.A.
Error (10^{-3} dmg/photon)	2	1	0.5	0.2	N.A.	N.A.	N.A.	N.A.	N.A.	N.A.

Table S5. Selected tetramer damage rates to approximate the effect of near-by Guanines to the formation of pyrimidine lesions.

Tetramer	μ_i $(10^{-3}\frac{dmg}{base})$	Tetramer	μ_i $(10^{-3}\frac{dmg}{base})$	Tetramer	μ_i $(10^{-3}\frac{dmg}{base})$
GTTG	4	GCCG	2	GCTG	2
GTTA	13	GCCA	4	GCTA	4
ATTG	14	ACCG	4	ACTG	3
ATTA	21	ACCA	5	ACTA	5

Software S1 (separate file). Monte-Carlo Software.

The file "SequenceData_PositionCorrelation_V1-22.llb" contains a complete data package for LabView 2014, which includes the software created in-house for carrying out the Monte Carlo simulation. Corresponding default values for a sample run are loaded automatically when the simulation is started. To calculate the UV sensitivities of a specific chronology, it must be added in the "Frequency analysis" tab in the "Pattern f. histogram" field in N codon pairs (an example chronology is already inserted). In the "Simulation of damage & synthesis of pools" tab, the corresponding N sequence pools can then be created by pressing the "Create pools synth cascade" button, each of which contains "# Sequences". After selecting the desired pools, they can be illuminated in silico by pressing the "Illuminate & Replicate pool" button.The context-dependent damage rates are saved in the "QE" field, the dose is specified in "Illumination settings".

SI References

(1) Ranjan, S.; Sasselov, D. D. Constraints on the Early Terrestrial Surface UV Environment Relevant to Prebiotic Chemistry. *Astrobiology* **2017**, *17* (3), 169–204. https://doi.org/10.1089/ast.2016.1519.

(2) Tataurov, A. V.; You, Y.; Owczarzy, R. Predicting Ultraviolet Spectrum of Single Stranded and Double Stranded Deoxyribonucleic Acids. *Biophys Chem* **2008**, *133* (1–3), 66–70. https://doi.org/10.1016/j.bpc.2007.12.004.

(3) Lemaire, D. G.; Ruzsicska, B. P. Quantum Yields and Secondary Photoreactions of the Photoproducts of dTpdT, dTpdC and dTpdU. *Photochemistry and Photobiology* **1993**, *57* (5), 755–769. https://doi.org/10.1111/j.1751-1097.1993.tb09207.x.

(4) Kumar, S.; Joshi, P. C.; Sharma, N. D.; Bose, S. N.; Jeremy, R.; Davies, H.; Takeda, N.; McCloskey, J. A. Adenine Photodimerization in Deoxyadenylate Sequences: Elucidation of the Mechanism through Structural Studies of a Major d(ApA) Photoproduct. *Nucleic Acids Research* **1991**, *19* (11), 2841–2847.

(5) Law, Y. K.; Azadi, J.; Crespo-Hernández, C. E.; Olmon, E.; Kohler, B. Predicting Thymine Dimerization Yields from Molecular Dynamics Simulations. *Biophysical Journal* **2008**, *94* (9), 3590–3600. https://doi.org/10.1529/biophysj.107.118612.

(6) Sztumpf, E.; Shugar, D. Photochemistry of Model Oligo- and Polynucleotides VI. Photodimerization and Its Reversal in Thymine Dinucleotide Analogues. *Biochimica et Biophysica Acta (BBA) - Specialized Section on Nucleic Acids and Related Subjects* **1962**, *61* (4), 555–566. https://doi.org/10.1016/0926-6550(62)90107-x.

(7) Johns, H. E.; Pearson, M. L.; LeBlanc, J. C.; Helleiner, C. W. The Ultraviolet Photochemistry of Thymidylyl-(3′5′)-Thymidine. *Journal of molecular biology* **1964**, *9* (2), 503-IN1. https://doi.org/10.1016/s0022-2836(64)80223-0.

(8) Kufner, C. L.; Krebs, S.; Fischaleck, M.; Philippou-Massier, J.; Blum, H.; Bucher, D. B.; Braun, D.; Zinth, W.; Mast, C. B. Sequence Dependent UV Damage of Complete Pools of Oligonucleotides. *Sci Rep* **2023**, *13* (1), 2638. https://doi.org/10.1038/s41598-023-29833-0.

(9) Kaszubowski, J. D.; Trakselis, M. A. Beyond the Lesion: Back to High Fidelity DNA Synthesis. *Front Mol Biosci* **2021**, *8*, 811540. https://doi.org/10.3389/fmolb.2021.811540.

(10) Kufner, C. L.; Zinth, W.; Bucher, D. B. UV-Induced Charge-Transfer States in Short Guanosine-Containing DNA Oligonucleotides. *Chembiochem : a European journal of chemical biology* **2020**. https://doi.org/10.1002/cbic.202000103.

(11) Bucher, D. B.; Kufner, C. L.; Schlueter, A.; Carell, T.; Zinth, W. UV-Induced Charge Transfer States in DNA Promote Sequence Selective Self-Repair. *Journal of the American Chemical Society* **2016**, *138* (1), 186–190. https://doi.org/10.1021/jacs.5b09753.

(12) Schreier, W. J.; Gilch, P.; Zinth, W. Early Events of DNA Photodamage. *Annual review of physical chemistry* **2015**, *66*, 497–519. https://doi.org/10.1146/annurev-physchem-040214-121821.

(13) Banyasz, A.; Douki, T.; Improta, R.; Gustavsson, T.; Onidas, D.; Vayá, I.; Perron, M.; Markovitsi, D. Electronic Excited States Responsible for Dimer Formation upon UV Absorption Directly by Thymine Strands: Joint Experimental and Theoretical Study. *Journal of the American Chemical Society* **2012**, *134* (36), 14834–14845. https://doi.org/10.1021/ja304069f.

(14) Trifonov, E. N. The Triplet Code from First Principles. *J. Biomol. Struct. Dyn.* **2004**, *22* (1), 1–11.

(15) Trifonov, E. N. Consensus Temporal Order of Amino Acids and Evolution of the Triplet Code. *Gene* **2000**, *261* (1), 139–151. https://doi.org/10.1016/s0378-1119(00)00476-5.

(16) Haig, D.; Hurst, L. D. A Quantitative Measure of Error Minimization in the Genetic Code. *J Mol Evol* **1991**, *33* (5), 412–417. https://doi.org/10.1007/BF02103132.

(17) Tze-Fei Wong, J. Coevolution of Genetic Code and Amino Acid Biosynthesis. *Trends in Biochemical Sciences* **1981**, *6*, 33–36. https://doi.org/10.1016/0968-0004(81)90013-x.

(18) Papentin, F. On Order and Complexity. II. Application to Chemical and Biochemical Structures. *Journal of Theoretical Biology* **1982**, *95* (2), 225–245. https://doi.org/10.1016/0022-5193(82)90241-7.

(19) Dufton, M. J. Genetic Code Synonym Quotas and Amino Acid Complexity: Cutting the Cost of Proteins? *J Theor Biol* **1997**, *187* (2), 165–173. https://doi.org/10.1006/jtbi.1997.0443.
(20) Arques, D. G.; Michel, C. J. A Complementary Circular Code in the Protein Coding Genes. *J Theor Biol* **1996**, *182* (1), 45–58. https://doi.org/10.1006/jtbi.1996.0142.
(21) Brooks, D. J.; Fresco, J. R. Increased Frequency of Cysteine, Tyrosine, and Phenylalanine Residues since the Last Universal Ancestor. *Mol Cell Proteomics* **2002**, *1* (2), 125–131. https://doi.org/10.1074/mcp.m100001-mcp200.
(22) Brooks, D. J.; Fresco, J. R.; Lesk, A. M.; Singh, M. Evolution of Amino Acid Frequencies in Proteins Over Deep Time: Inferred Order of Introduction of Amino Acids into the Genetic Code. *Molecular Biology and Evolution* **2002**, *19* (10), 1645–1655. https://doi.org/10.1093/oxfordjournals.molbev.a003988.
(23) Eck, R. V.; Dayhoff, M. O. Evolution of the Structure of Ferredoxin Based on Living Relics of Primitive Amino Acid Sequences. *Science* **1966**, *152* (3720), 363–366. https://doi.org/10.1126/science.152.3720.363.
(24) Xia, T.; SantaLucia, J., Jr.; Burkard, M. E.; Kierzek, R.; Schroeder, S. J.; Jiao, X.; Cox, C.; Turner, D. H. Thermodynamic Parameters for an Expanded Nearest-Neighbor Model for Formation of RNA Duplexes with Watson-Crick Base Pairs. *Biochemistry* **1998**, *37* (42), 14719–14735. https://doi.org/10.1021/bi9809425.
(25) Osawa, S.; Jukes, T. H.; Watanabe, K.; Muto, A. Recent Evidence for Evolution of the Genetic Code. *Microbiol Rev* **1992**, *56* (1), 229–264. https://doi.org/10.1128/mr.56.1.229-264.1992.
(26) Crick, F. H.; Brenner, S.; Klug, A.; Pieczenik, G. A Speculation on the Origin of Protein Synthesis. *Orig Life* **1976**, *7* (4), 389–397. https://doi.org/10.1007/BF00927934.
(27) Eigen, M.; Schuster, P. The Hypercycle. *Die Naturwissenschaften* **1978**, *65* (1), 7–41. https://doi.org/10.1007/bf00420631.
(28) Eigen, M.; Gardiner, W.; Schuster, P.; Winkler-Oswatitsch, R. The Origin of Genetic Information. *Sci Am* **1981**, *244* (4), 88–92, 96, et passim. https://doi.org/10.1038/scientificamerican0481-88.
(29) Ferreira, R.; Coutinho, K. R. Simulation Studies of Self-Replicating Oligoribotides, with a Proposal for the Transition to a Peptide-Assisted Stage. *J Theor Biol* **1993**, *164* (3), 291–305. https://doi.org/10.1006/jtbi.1993.1155.
(30) Nelsestuen, G. L. Amino Acid-Directed Nucleic Acid Synthesis. A Possible Mechanism in the Origin of Life. *Journal of Molecular Evolution* **1978**, *11* (2), 109–120. https://doi.org/10.1007/bf01733887.
(31) Möller, W.; Janssen, G. M. C. Transfer RNAs for Primordial Amino Acids Contain Remnants of a Primitive Code at Position 3 to 5. *Biochimie* **1990**, *72* (5), 361–368. https://doi.org/10.1016/0300-9084(90)90033-d.
(32) Chaley, M. B.; Korotkov, E. V.; Phoenix, D. A. Relationships among Isoacceptor tRNAs Seems to Support the Coevolution Theory of the Origin of the Genetic Code. *J Mol Evol* **1999**, *48* (2), 168–177. https://doi.org/10.1007/pl00006455.
(33) Henikoff, S.; Henikoff, J. G. Amino Acid Substitution Matrices from Protein Blocks. *Proc Natl Acad Sci U S A* **1992**, *89* (22), 10915–10919. https://doi.org/10.1073/pnas.89.22.10915.
(34) Eigen, M.; Winkler-Oswatitsch, R. Transfer-RNA, an Early Gene? *Naturwissenschaften* **1981**, *68* (6), 282–292. https://doi.org/10.1007/BF01047470.
(35) Kvenvolden, K. A.; Lawless, J. G.; Ponnamperuma, C. Nonprotein Amino Acids in the Murchison Meteorite. *Proc Natl Acad Sci U S A* **1971**, *68* (2), 486–490. https://doi.org/10.1073/pnas.68.2.486.
(36) Riddle, D. S.; Santiago, J. V.; Bray-Hall, S. T.; Doshi, N.; Grantcharova, V. P.; Yi, Q.; Baker, D. Functional Rapidly Folding Proteins from Simplified Amino Acid Sequences. *Nat Struct Biol* **1997**, *4* (10), 805–809. https://doi.org/10.1038/nsb1097-805.
(37) Yarus, M. Specificity of Arginine Binding by the Tetrahymena Intron. *Biochemistry* **1989**, *28* (3), 980–988. https://doi.org/10.1021/bi00429a010.
(38) Hartman, H. Speculations on the Origin of the Genetic Code. *J Mol Evol* **1995**, *40* (5), 541–544. https://doi.org/10.1007/BF00166623.
(39) Taylor, F. J. R.; Coates, D. The Code within the Codons. *Biosystems* **1989**, *22* (3), 177–187. https://doi.org/10.1016/0303-2647(89)90059-2.

(40) Ikehara, K.; Omori, Y.; Arai, R.; Hirose, A. A Novel Theory on the Origin of the Genetic Code: A GNC-SNS Hypothesis. *J Mol Evol* **2002**, *54* (4), 530–538. https://doi.org/10.1007/s00239-001-0053-6.
(41) Baumann, U.; Oro, J. Three Stages in the Evolution of the Genetic Code. *Biosystems* **1993**, *29* (2–3), 133–141. https://doi.org/10.1016/0303-2647(93)90089-u.
(42) Li, D. J. Formation of the Codon Degeneracy during Interdependent Development between Metabolism and Replication. *Genes* **2021**, *12* (12), 38. https://doi.org/10.3390/genes12122023.
