# Supplementary material for: Selection of Early Life Codons by Ultraviolet Light
Source: ACS Cent Sci. 2025 Jan 8;11(1):147–56. doi: 10.1021/acscentsci.4c01623 (PMC11758376; doi:10.1021/acscentsci.4c01623)
Supplement: Supplementary file 3 — oc4c01623_si_003.pdf [file oc4c01623_si_003.pdf]

oc-2024-01623u.R1

Name: Peer Review Information for "Selection of Early Life Codons by Ultraviolet Light"

First Round of Reviewer Comments

Reviewer: 1

Comments to the Author

The manuscript by Kufner et. al. describes the modeling of UV stability of random 150-mer sequences of single stranded DNA. The input data for the modeling had previously been generated experimentally by some of the same authors. The authors calculated the stability of 150-mers for a numerous potential 'protogenomes', which were randomly assembled 'in silico' from different subsets of codons. Furthermore, the authors defined and calculated the metric of accumulated UV stability, which was used to compare the impact of UV irradiation on different hypothetical codon chronologies, starting from a protogenome made from only a single codon to the full genome of all 64 codons.

This study demonstrated that the identity of the first ~4 codons incorporated into a growing protogenome most crucially determined the UV-resistance of the entire chronology of codon incorporation. Most importantly, the results from the selection for UV-resistance described in this manuscript correlated well with previously published models of the genetic code evolution that only considered numerous other properties and conditions, but not UV damage.

The manuscript was well-written and easy to follow.

Comments and suggestions:

To clarify the content of this manuscript in the abstract, the phrase "utilizing sequence-context-dependent damage rates determined by high throughput sequencing experiments" should be modified to read "utilizing sequence-context-dependent damage rates previously determined by high throughput sequencing experiments".

In Fig. 1, The acronyms 2 PpB, and M (as in small M) need to be explained.

In Fig. 2, sub-3mers and sub 3-mer are mentioned. What does that refer to?

The phrase "product of series" in Fig. 3 is unclear and needs to be explained.

The reference callout "(36)" is unformatted and not pointing to the correct article.

References 37 and 38 specifically, and probably all references, should be double-checked for accurateness and usefulness.

Author's Response to Peer Review Comments:

Answer to referee comments are green. Changes in the manuscript text are blue.

REVIEWER COMMENTS

Reviewer: 1

Recommendation: Publish in ACS Central Science after minor revisions noted.

Comments:
The manuscript by Kufner et. al. describes the modeling of UV stability of random 150-mer sequences of single stranded DNA. The input data for the modeling had previously been generated experimentally by some of the same authors. The authors calculated the stability of 150-mers for a numerous potential 'protogenomes', which were randomly assembled 'in silico' from different subsets of codons. Furthermore, the authors defined and calculated the metric of accumulated UV stability, which was used to compare the impact of UV irradiation on different hypothetical codon chronologies, starting from a protogenome made from only a single codon to the full genome of all 64 codons.
This study demonstrated that the identity of the first ~4 codons incorporated into a growing protogenome most crucially determined the UV-resistance of the entire chronology of codon incorporation. Most importantly, the results from the selection for UV-resistance described in this manuscript correlated well with previously published models of the genetic code evolution that only considered numerous other properties and conditions, but not UV damage. The manuscript was well-written and easy to follow.

We thank the Referee for this positive assessment of the work.

Comments and suggestions:

To clarify the content of this manuscript in the abstract, the phrase "utilizing sequence-contextdependent damage rates determined by high throughput sequencing experiments" should be modified to read "utilizing sequence-context-dependent damage rates previously determined by high throughput sequencing experiments".

The requested change has now been made in the summary of the manuscript.

In Fig. 1, The acronyms 2 PpB, and M (as in small M) need to be explained.

We agree that an explanation is needed here and have now added this to the caption for Figure 1: "The irradiation dose is stated in PpB, i.e., the photons per base."
To make it clear that M refers to the steps used in Figure 1C, we have changed the caption accordingly:

"In simple proto-lifeforms, only a few amino acids and a small part of the genetic code were used early on (step number M is small). Only proto-genomes using UV-stable codons would survive here. Throughout its development, newly added codons at higher step numbers M change the UV stability of the protogenome until all 32 codon pairs (64 triplets) are used in the genome."

In Fig. 2, sub-3mers and sub 3-mer are mentioned. What does that refer to?

We thank the Referee for pointing out this inconsistency. We have now changed the axis labelling in Fig. 2C and the caption of Figure 2 so that the 3-mers (no longer sub-3-mers) of Fig. 2A is always referred to for the sake of clarity. In the caption for Figure 3C, we write:

"**(C)** Damage rates of selected 3mers, shown in (A), e.g., GGG, GGC, GTC."

The phrase "product of series" in Fig. 3 is unclear and needs to be explained.

We agree that the description of the accumulated UV susceptibility is insufficient here. We have therefore amended the caption for Figure 3B as follows:

"The x-axis is scaled logarithmically, as the accumulated UV stability, $\chi_{acc,N=32}(C)=\prod_{M=1}^{N=32}\chi_M$, is obtained by multiplying the UV stabilities of the individual steps and, therefore, spans many orders of magnitude."

The reference callout "(36)" is unformatted and not pointing to the correct article.
References 37 and 38 specifically, and probably all references, should be double-checked for accurateness and usefulness.

We thank the Referee for pointing out the orphan reference. We have removed it and checked the entire manuscript again for the correctness of the references.
